# A Comprehensive Review of the Functionalized Integrated Application of Gel Polymer Electrolytes in Electrochromic Devices

**DOI:** 10.1007/s40820-025-01909-8

**Published:** 2026-01-09

**Authors:** Lei Xu, Leipeng Zhang, Dongqi Liu, Zichen Ren, Wenchao Liu, Yike Zhang, Yuqiang Wang, Jiawu Sun, Rui Yang, Zekuo Lv, Jiupeng Zhao, Yao Li

**Affiliations:** 1 School of Chemistry and Chemical Engineering, Harbin Institute of Technology, Harbin, 150001 People’s Republic of China; 2 Center for Composite Materials and Structure, Harbin Institute of Technology, Harbin, 150001 People’s Republic of China

**Keywords:** Gel polymer electrolytes, Electrochromic devices, Multifunctional gels, Polymer designs

## Abstract

With the global push for energy conservation and the rapid development of low-power, flexible and wearable optical displays, the demand for electrochromic technology has surged. Gel polymer electrolytes (GPEs), a crucial component of electrochromic devices (ECDs), show great promise in applications. This is attributed to their efficient ion-transport capabilities, excellent mechanical properties and strong adhesion. All of these characteristics are conducive to enhancing the safety of the devices, streamlining the packaging process, significantly improving the electrochromic performance of ECDs and boosting their commercial application potential. This review provides a comprehensive overview of GPEs for ECDs, focusing on their basic designs, functional modifications and practical applications. Firstly, this review outlines the fundamental design of GPEs for ECDs, encompassing key performance index, classification, gelation mechanism and preparation methods. Building on this foundation, it provides an in-depth discussion of functionalized GPEs developed to enhance device performance or expand functionality, including electrochromic, temperature-responsive, photo-responsive and stretchable self-healing GPE. Furthermore, the integration of GPEs into various ECD applications, including smart windows, displays, energy storage devices and wearable electronic, are summarized to highlight the advantages that the design of GPEs brings to the practical application of ECDs. Finally, based on the summary of GPEs employed for ECDs, the challenges and development expectations in this direction were indicated.

## Highlights

- In response to the demands of electrochromic devices, the advantages and designs of the corresponding multifunctional integrated gel polymer electrolytes were discussed.
- Through reviewing the applications of electrochromic devices based on gel polymer electrolytes, the remarkable advantages that gel polymer electrolytes bring to electrochromic devices and their practical applications in electrochromic devices were analyzed.
- The future research directions of gel polymer electrolytes for electrochromic devices were explored, thereby facilitating their further development and commercial application.

## Graphical abstract

**Figure:**
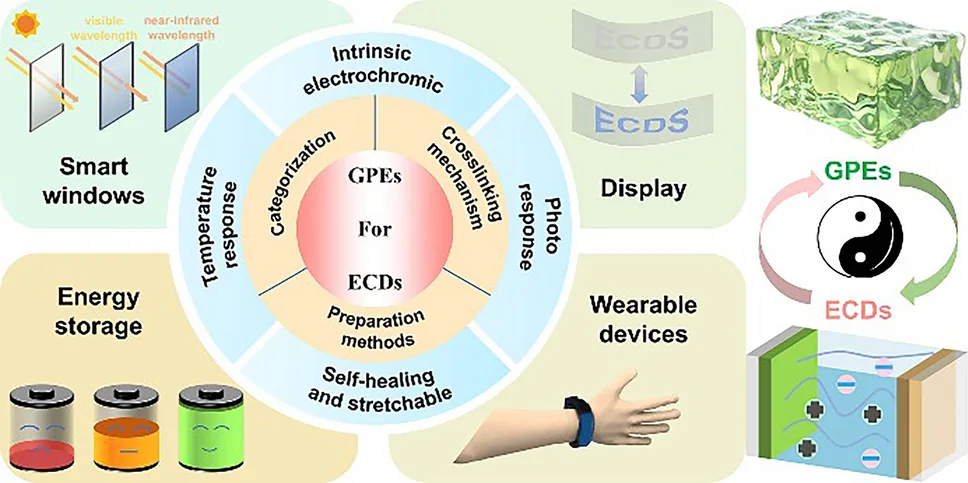

## Introduction

Electrochromism is a phenomenon whereby the molecular or lattice structure of electrochromic materials undergoes a reversible redox reaction under the action of an external electric field, thereby altering the optical properties of transmittance, reflectance and absorbance [1, 2]. Since electrochromism was first proposed in 1961 by Platt., electrochromic technology had experienced rapid development, attaining numerous significant accomplishments in both fundamental research and practical applications [3]. Electrochromic devices (ECDs) boast advantages such as low power consumption, fast response speed, reversibility and good stability and have thus been extensively utilized in multiple domains such as smart window, intelligent display and military camouflage [4–8]. Indeed, over the last three decades, an increasing amount of research endeavors have been dedicated to the synthesis of novel ECDs, and several commercial applications have emerged.

**Fig. 1:**
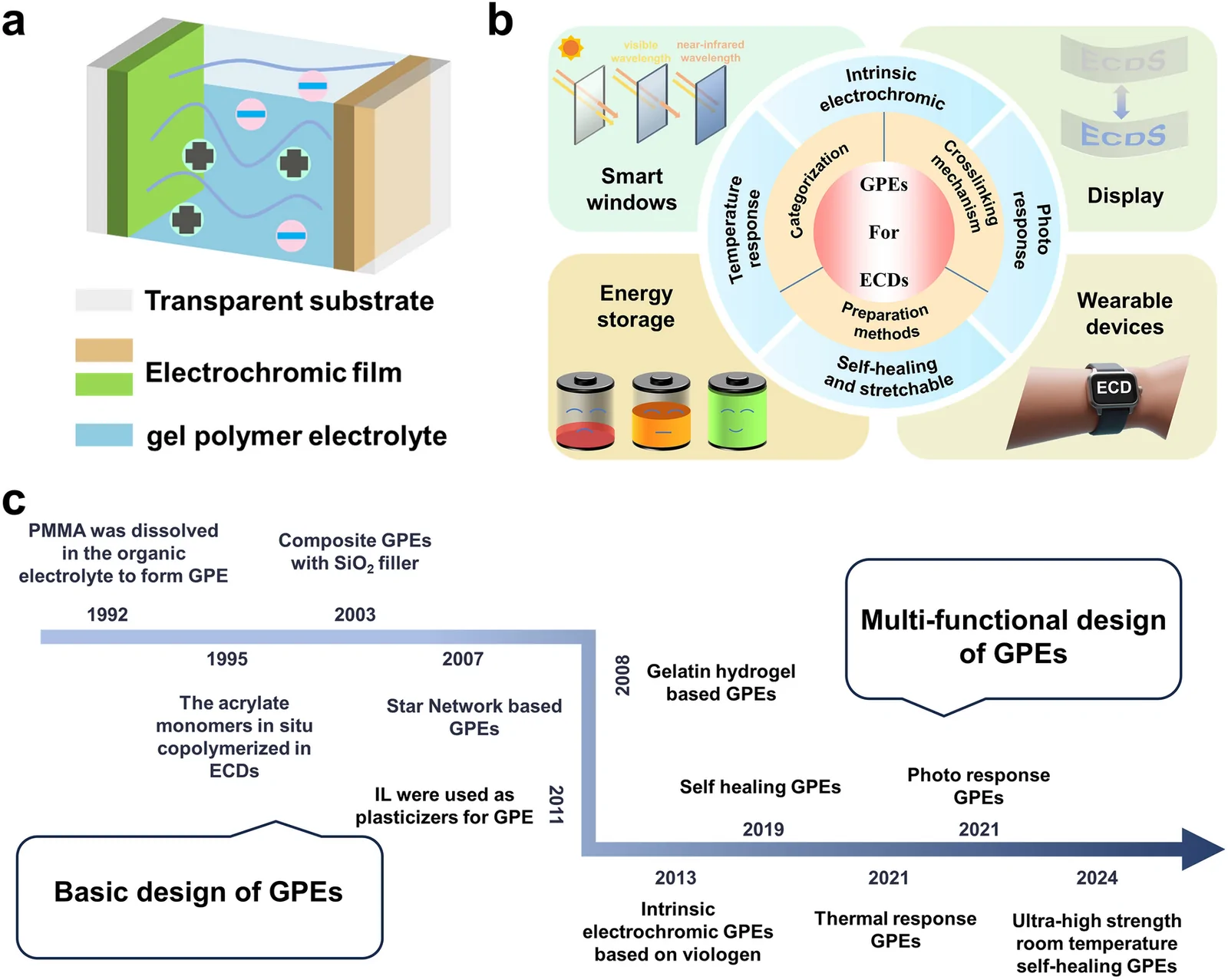
**a** Schematic diagram of a typical ECD structure. **b** GPEs for ECDs: material design, versatility and applications. **c** Developmental timeline of GPEs for ECDs [15–25]

ECDs are similar in structure to rechargeable batteries, using a sandwich-structured dual electrode system [9]. As shown in Fig. 1a, the conventional ECDs structure encompasses five components: two transparent conductive layers positioned at the ends of the device, an electrochromic layer, an electrolyte and an ion storage layer in the middle [1, 10]. Electrolyte layer, also called ion-transport layer, is used to provide an ionic connection between the electrodes, allowing ion exchange between the cathode and anode but avoiding direct electrical contact [9, 11]. In ECDs, the electrolyte determines the degree and speed of electrochemical reactions of electrochromic materials, which directly affects the electrochromic performance of ECDs. Liquid electrolytes possess relatively high ionic conductivity and exhibit superior coloring and bleaching efficacy, but are limited in application due to the risk of leakage [12]. Conversely, solid electrolytes have the advantage of high safety, but their slower ion diffusion rate limits the electrochromic performance of ECDs [13, 14]. And the high rigidity of inorganic solid electrolytes also limits their application in flexible wearable devices. To this end, gel polymer electrolytes (GPEs) with high ionic conductivity and safety, which combine the merits of both liquid and solid electrolytes and circumvent their drawbacks, have gained increasing popularity and are regarded as the ideal dielectric layers for ECDs.

GPEs consist of long-chain polymer, liquid plasticizers and metal salts. In GPEs, ion transport takes place simultaneously in the polymer and the liquid plasticizer [26]. Meanwhile, the liquid plasticizer reduces the crystallinity of the polymer chains and enhances the movement of the polymer's chain segments, thereby increasing the ionic conductivity of the GPEs [27]. The mechanical strength of GPEs is mainly supported by polymers, but it is still influenced by liquid plasticizers and electrolyte salts [28]. Therefore, the interactions among the polymer molecular chains, the liquid plasticizer and the electrolyte salt need to be fully considered when designing GPEs. Moreover, as a polymeric material with flexible design of physical and chemical properties, it can be endowed with functionalities through chemical modification and copolymerization modification, which implies that GPEs can be designed to achieve the desired functions [13, 29, 30]. In addition, functional substances such as viologen and TiO₂ can also be incorporated into GPEs to enhance their properties [31, 32]. Therefore, in addition to being the electrolyte layer of the ECDs, GPEs are also anticipated to further enhance the performance and expand the capabilities of ECDs. GPEs with viologen compound have electrochromic function themselves, acting as both electrolyte and electrochromic material in ECDs, thus simplifying the structure of the device [33, 34]. In addition, the dimerization of viologen can be effectively inhibited by the polymer gel, thus increasing the service life of the device. Polymer gels with thermal response function are selected as the electrolyte layer of ECDs to provide thermochromic function for ECDs, so as to prepare smart devices with both electrochromism and thermochromism [35, 36]. Except respond to temperature, some smart polymer gels with photo-responsive properties have been also reported as electrolyte layers of ECDs that can be used to reduce the driving voltage of ECDs and endow ECDs with function of photochromism [32, 37]. Self-healing and stretchable properties have also been introduced into GPEs for ECDs, which brings great advantages for the manufacture of wearable ECDs. Therefore, in addition to the electrochemical performance and mechanical stability as an electrolyte, the functionalization and intelligence of GPEs should also be focused in the design of ECDs.

Although relevant reviews have reported on the design essentials, development status and issues to be addressed regarding GPEs for ECDs, these reports are relatively outdated [38–40]. Moreover, there is a lack of a systematic review on the multifunctional integrated design of GPEs. In this review, we elaborate on the design criteria, functionalization and applications of GPEs for ECDs in various electrochromic devices, as depicted in Fig. 1b. Firstly, we systematically introduce the basic theory of GPE for ECDS, including the performance requirements, classification, gelation mechanism and preparation process of GPE. Subsequently, we collect and document the historical research achievements of GPEs in ECDs over the past three decades, as illustrated in Fig. 1c. Prior to 2011, the research on GPEs in ECDs primarily concentrated on the basic properties of GPE as an electrolyte layer. Since 2011, the research focus has shifted to functionalized GPEs to further improve the performance or enrich the functionality of ECDs. Therefore, on the basis of introducing the basic knowledge of GPEs, we particularly review and discuss the design and effects of intelligent functional GPEs, such as intrinsically electrochromic, temperature-responsive, photo-responsive, self-healing and stretchability. Based on the aforementioned content, we further summarize the application of GPEs in various classical electrochromic devices, including electrochromic smart windows, electrochromic energy storage devices, electrochromic displays and wearable ECDs. Finally, we put forward the challenges and future directions for further applications in the field of GPEs for ECDs.

## Basics of GPEs for ECDs

GPEs are commonly formed by adding liquid plasticizers and solvents to a polymer matrix to create a stable gel structure [41, 42]. Therefore, GPEs usually consist of polymer matrix, solvents serving as plasticizers, salts and some fillers [43, 44]. It can be categorized as a two-phase system composed of an ionic conductive medium encapsulated by the polymer matrix. GPEs possess both the cohesive properties of polymers and the diffusive transport properties of liquids due to their distinctive hybrid network structures. This means that the relative content of solid-phase polymers and liquid-phase plasticizers in GPEs is paramount importance for their mechanical stability and ionic conductivity [45, 46]. Owing to their distinctive properties, GPEs exhibit higher conductivity than solid polymer electrolytes, resulting in ECDs with better optical modulation and faster response time. While these electrolytes exhibit higher conductivity, they generally have inferior mechanical properties. Poor mechanical properties of GPEs may lead to short-circuiting of ECDs, resulting in device failure and raising safety issues and rendering them unsuitable for use in certain preparation processes [9]. In practical applications, a trade-off needs to be established between the enhancement of ionic conductivity and the degradation of mechanical strength. Furthermore, GPEs should possess high stability, including voltage stability, temperature stability and stability to electrochromic materials and their interfaces. Finally, the optical transmission of GPEs should also be focused on and not adversely affect the electrochromic effect of ECDs.

GPEs, serving as a crucial component of ECDs, constitute interdisciplinary scientific research. And for the successful applications of GPEs in ECDs, the following requirements need to be fulfilled [9, 41, 47, 48].

*High ionic conductivity:* GPEs, as electrolytes in electrochemical systems, must initially meet high ionic conductivity and electronic insulating, which can facilitate ion transport and minimize self-discharge of devices. GPEs with high ionic conductivity enable ECDs with high optical modulation and fast response time.

*Wide potential window:* The GPEs is required to possess a broad potential window, thereby enabling it to withstand higher or lower potentials when a voltage is imposed, with the aim of attaining controllable color variations. This is attributed to the fact that the electrochromic device (ECD) needs to accomplish oxidation–reduction reactions at diverse potentials, and the GPE is supposed to be capable of tolerating the applied potential as demanded.

*Chemical and electrochemical stability:* GPEs must be resistant to corrosion and decomposition in electrochemical reactions to ensure their reliability during prolonged periods of usage. This includes maintaining the chemical stability of both the electrode materials and the electrolyte itself.

*High optical transparency:* GPEs require to have high optical transparency to guarantee that the ECD has a relatively high optical regulation threshold.

*Good mechanical strength:* The superior mechanical strength of the GPE enhances the external forces resistance of ECDs. For flexible wearable ECDs, GPEs should exhibit mechanical properties such as high tenacity and stretchability.

*High adhesion:* The high adhesion of GPEs ensures they are in close contact with the electrochromic material and reduce the interfacial resistance. In addition, GPEs with high adhesion can simplify the preparation process of ECDs and prevent the device delamination.

*Environmental friendliness and low cost:* Low costs and environmental friendliness are highly desirable for the successful commercialization and implementation of the electrochromic devices. Hence, the polymer electrolyte should be inexpensive, environmental friendly and readily available.

### Classification of GPEs for ECDs

Although the GPEs are present in solid state, the liquid phase is still the main component of the GPEs [49]. Depending on the type of plasticizer, GPEs are classified as hydrogels, organogels and ionogels.

#### Hydrogel Electrolyte

Aqueous plasticizer has advantages of low cost, safety, environmental friendliness and high salt solubility, making hydrogels to be one of the commonly used GPEs in ECDs [50]. Typically, hydrogels require polymers with a high number of hydrophilic groups such as carboxyl groups (–COOH), hydroxyl groups (–OH), carbonyl groups (–C═O) and amino groups (–NH_2_), thus locking water in the hydrogel electrolyte [51, 52]. Polyvinyl alcohol (PVA) is widely used as a polymer framework for hydrogels due to its large number of hydroxyl groups. Lu et al. used PVA as the polymer chain of the hydrogel to prepare 3D printable hydrogel electrolytes for privately customizable flexible ECDs [53]. Flexible ECDs based on this hydrogel electrolyte exhibit high optical contrast (max. 54.4% at 360 nm), excellent cycling stability (< 5% reduction after 10,000 s), and an optical contrast reduction of less than 19% after 5000 bending cycles, which offers great potential for application. Cellulose-based materials chemically modified to introduce hydrophilic functional groups are also one of the common polymer chains for hydrogels. Xiao et al. prepared a hydrogel electrolyte using sodium carboxymethyl cellulose (CMC-Na). CMC-Na can be regarded as a polyelectrolyte that provides excellent ionic transport in addition to mechanical support for hydrogels [54]. Upon complexation with 1,1′-bis (2-sulfonatoethyl) viologen (SEV) or 1,1′-bis (3-sulfonatopropyl) viologen (SPV), ECDs based on this hydrogel exhibit excellent optical modulation and cycling stability. Compared to PVA and cellulose-based materials, polyacrylamide-based hydrogel electrolytes have gained popularity due to their in situ polymerization in ECDs. Gao et al. prepared hydrogel electrolytes for ECDs using PAM as the polymer chain and ZnCl_2_ as the conducting salt [55]. The hydrogel electrolyte possesses high ionic conductivity and transparency, and can be polymerized in situ in ECDs, resulting in excellent electrochromic properties of ECDs. Although the hydrogel has good performance in ECDs, the high freezing point and low boiling point of water limits the application of hydrogel electrolytes at low temperatures [51, 56, 57]. More importantly, hydrogel electrolyte-based ECDs must operate at lower voltages to avoid water decomposition, which makes it difficult for some electrochromic materials to undergo adequate electrochemical reactions [58].

#### Organogel Electrolyte

In comparison with hydrogel electrolytes, organic GPEs that utilize organic solvents with high dielectric constants as plasticizers provide ECDs with wider potential window and operating temperature, thus further improving the performance of ECDs [59, 60]. Propylene carbonate (PC) is one of the most commonly used plasticizers for GPEs due to its high dielectric constant and lithium salt solubility. Jakub Reiter et al. fabricated polymer GPEs based on poly(ethyl methacrylate) (PEMA) and poly(2-ethoxyethyl methacrylate) (PEOEMA) with entrapped solutions of lithium perchlorate in PC by direct UV-initiated polymerization [61]. The high ionic conductivity of the GPEs, coupled with a high potential window of 2.5 V and thermal stability at 125 °C, bestows excellent electrochromic properties and stability upon the ECD. The viscosity of the plasticizer is also one of the important factors affecting the ionic conductivity of GPEs. In another study, Gregory A. Sotzing et al. prepared GPEs using PC, ethylene carbonate (EC) and diethyl carbonate (DEC) as cosolvents and poly (ethylene glycol) diacrylate (PEGDA) as polymer chain, and balancing the dielectric constant and viscosity of plasticizers to improve the ionic conductivity of GPEs was explored [62]. The optical modulation of ECDs was improved by 14% by using a cosolvent of PC and DEC as a plasticizer for GPEs. Nevertheless, the inferior safety and volatility of organic plasticizers can exert a detrimental impact on ECDs [63].

#### Ionogel Electrolyte

Ionic liquids (ILs), also known as room-temperature molten salts, possess advantages such as negligible vapor pressure, high temperature stability and a large electrochemical stability window, thus becoming one of the ideal plasticizers for GPEs used in ECDs [64, 65]. IL-based GPEs effectively ameliorate the shortcomings of organic GPEs, such as flammable and volatile, thus being regarded as promising GPEs. Lu et al. reported a P(VDF-HFP) film with SiO_2_ growing on the surface (SiO_2_-on-P (VDF-HFP)) [44]. And it was immersed in LiClO_4_/1-butyl-3-methylimidazolium tetrafluoroborate (BMIMBF_4_) to prepare the ionogel electrolyte for ECDs. The presence of SiO_2_ significantly enhanced the ion dissociation in GPEs and thus enhanced the performance of ECDs. Although IL offers significant advantages as a plasticizer for GPEs, its high viscosity limits the ionic transport of GPEs [66]. Therefore, ILs and organic solvents are usually chosen together as plasticizers for GPEs to mitigate the adverse effect of high viscosity of ILs on ionic conductivity [67]. Wang et al. used a mixture plasticizer of PC and 1-ethyl-3-methylimidazolium tetrafluoroborate (EMIMBF_4_) to prepare the GPE [68]. The use of the cosolvent strategy significantly enhances the electrochemical properties of GPEs, thus conferring better electrochromic properties to ECDs. Deep eutectic solvents (DES), which can be regarded as a type of ILs, have the advantages of inexpensive and environmentally friendly in addition to the excellent performance of ILs [69, 70]. Zhao et al. prepared a GPE for ECDs with a wide temperature range by choosing LiTFSI in N-methylacetamide (NMA) as DES plasticizer [63]. Benefiting from the rational design of this EDS-based GPE, the ECD exhibits good electrochromic performance over a wide temperature range.

The advantages and disadvantages, the material as well as performance comparisons of hydrogels, organogels and ionogels are summarized in Table 1

**Table 1:** Comparison of advantages and disadvantages, the material and performance of hydrogels, organogels and ionogels

Classification of GPEs	Advantages	Drawbacks	Plasticize	Polymer	Salts	Optical modulation	Response time	Cyclic stability	References
Hydrogels	High ionic conductivity Inexpensive Environmentally friendly	Narrow electrochemical window Easy to solidify and volatile	water	PVA	Li Cl	54.4% (max at 360 nm)	10.4 s/15.3 s	10,000 s	[53]
			water	CMC-Na	CMC-Na	SEV: 55.75% SPV: 55.98%	Within 3 s	SEV: 1000 SPV: 8000	[54]
			water	PAM	ZnCl_2_	55.90%	11 s/10 s	9200	[55]
Organogels	Wide potential window Good low temperature performance	Volatile and flammable Unenvironmentally	PC	PEOEMA and PEMA	LiClO_4_	40%-45%	20 s-25 s	No mentioned	[61]
			PC/EC/DEC	PEGDA	LiTrif	50%	No mentioned	No mentioned	[62]
Ionogels	Wide potential window Non-volatile	Expensive Poor ion mobility	BMIMBF_4_	SiO_2_-on-P(VDF-HFP)	LiClO_4_	72%	No mentioned	No mentioned	[44]
			PC/EMIMTFSI	PMMA	LiClO_4_	71%	62.6 s/41.2 s	No mentioned	[68]
			NMA + Li TFSI	P(DEEA-co-IOBA)	LiTFSI	45%	8.4 s/11.4 s	100	[63]

### Gelation Mechanism and Preparation Process of GPEs

Typically, the gelation of GPEs is formed by the three-dimensional (3D) polymer network. The polymer backbone of GPEs for ECDs is generally expected to require the following characteristics: (i) highly flexible molecular chains and low crystallinity (or amorphous polymer chains); (ii) polar functional groups to facilitate salt dissociation; and (iii) high molecular weight. This section focuses on the gelation mechanism and preparation technology of GPEs for ECDs. First of all, the interactions between the polymer chains that trigger the gelation of GPEs are outlined, including physical entanglement and cross-linking between the polymer chains. Subsequently, the preparation techniques are classified and summarized based on whether the GPEs are gelated in situ.

#### Gelation Mechanism of GPEs

**Fig. 2:**
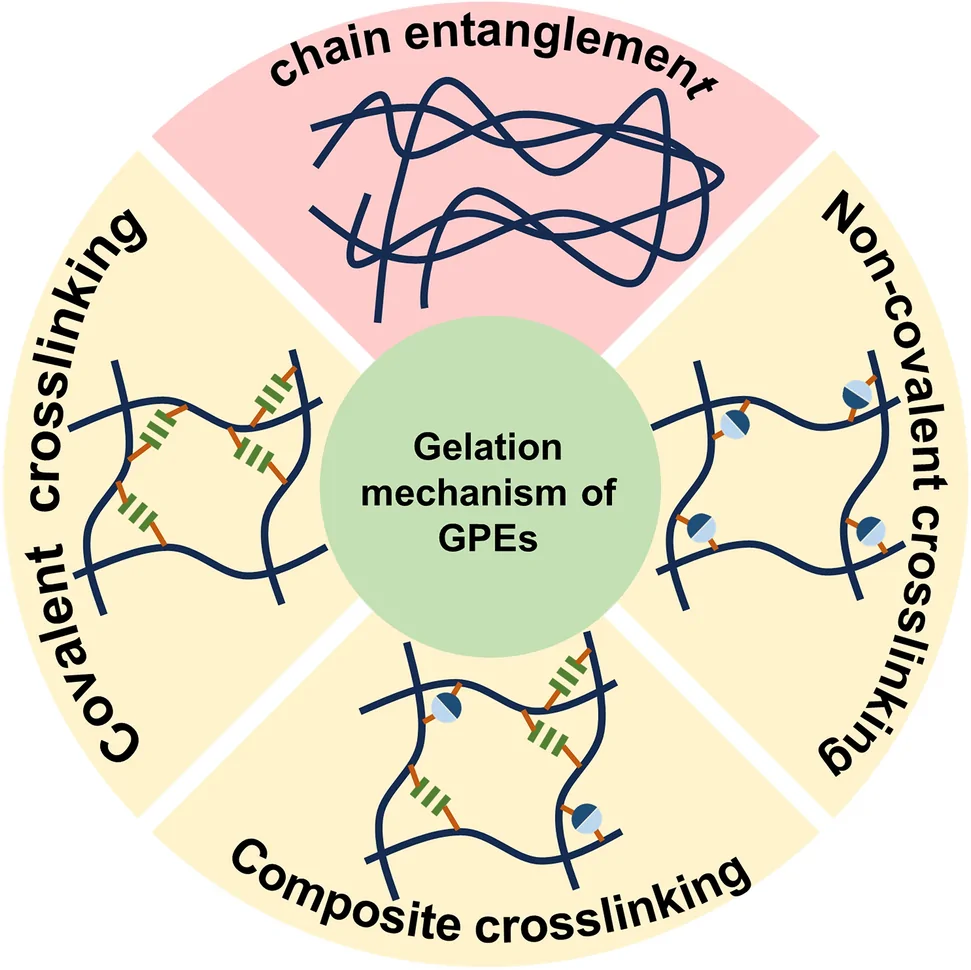
Gelation mechanism of GPEs

Polymer gels are conformal systems in which a good solvent dissolves a three-dimensional network of polymers. Thus, the polymer can be viewed as a gelling agent for the GPEs, supporting the mechanical strength of the GPEs. As shown in Fig. 2, the gelation mechanism of GPEs involves the interaction of polymer chains to form a three-dimensional network, primarily classified into chain entanglement and cross-linking.

##### The Entanglement of Polymer Chains

The significant difference between polymers and other materials is that they contain fairly long and flexible macromolecules, with polymer chains constructed from long chains that become entangled with each other, a fundamental feature in the physical network of polymers [71]. Since gelation of GPEs is produced by polymers, polymer chain entanglement is inevitably present in any GPEs. The presence of long-chain entanglements significantly affects the mechanical properties of the polymer, especially toughness and strength. In order to ensure the mechanical strength required for ECD, the GPEs need to have a high polymer concentration to achieve the strong chain entanglement [72]. Tan et al. reported a eutectic gel based on the physical entanglement of polymer chains [73]. Owing to the gelation mechanism that relies on complete physical entanglement of polymer chains, this eutectic gel exhibits excellent properties, including mechanical robustness, self-healing and self-adhesion. In addition to its impact on mechanical properties, the entanglement of polymer chains also influences ion transport in GPEs. Guo et al. designed a hydrogel electrolyte with highly entangled polymer chains to enhance the charge–discharge rate of aqueous zinc-ion batteries [74]. This hydrogel electrolyte enables the rapid and stable migration of Zn^2^⁺ ions while withstanding high current densities benefit from the strong entanglement between adjacent polymer chains, thereby advancing the development of quasi-solid-state batteries for fast charge–discharge applications. Although the mechanical properties and ion transport of GPEs have been improved through reasonable regulation of chain entanglement, there is still a lack of relevant research on their reuse in GPEs for ECDs, which may be one of the future research directions.

##### Crosslinking Reaction of Polymer Chains

In this case, in addition to the entanglement of the polymer chains, the gelation of GPEs for ECDs is further enhanced by the cross-linking reaction between the molecular chains of the polymer. Based on the types of cross-linking chemical bonds, the cross-linking of gels can be categorized into covalent cross-linking, non-covalent cross-linking and composite cross-linking of covalent and non-covalent bonds.

Covalent bonds employed for cross-linking GPEs are classified as permanent covalent bonds and dynamic (reversible) covalent bonds based on whether they can be reorganized upon breakage. From the perspective of high modulus and mechanical strength, permanent covalent bond cross-linking is attractive for GPEs [75, 76]. Hence, permanent covalent bonds are extensively utilized for cross-linking of GPEs for ECDs. Pooi See Lee et al. adopted PEGDA as the covalent bond cross-linker of GPEs to enhance the strength of GPEs [42]. Although the formation of 3D polymer networks through the formation of permanent covalent bonds between polymer chains is a straightforward approach to prepare GPEs, covalent bond cross-linking results in intensified brittleness of the GPE, reduces its stretchability and renders the GPEs incapable of self-repairing (healing) due to the irreversibility of the permanent covalent bonds [77, 78]. In contrast, the cross-linking structure formed by dynamic covalent and non-metric bonds (hydrogen bonding, charge interactions and van der Waals interactions) enables the GPE to possess favorable stretchability and self-repairing properties due to the weak and reversible nature of dynamic covalent and non-metric bonds [77, 79–81]. Liu et al. developed a novel supramolecular GPE by introducing non-covalent supramolecular self-assembly networks into polymer networks in the presence of environmentally friendly and cost-effective DES [82]. The GPEs exhibit high tensile properties, self-healing ability, ultra-fast in situ underwater and low-temperature adhesion, providing a promising strategy for the development of functionally integrated ECDs with good mechanical properties. In addition, there are several strategies that integrate covalent and non-covalent cross-linking to obtain GPEs with both high strength and self-healing capabilities. Zhao et al. selected PEGDA as a covalent bond cross-linker to furnish high strength of GPEs and concurrently exploited the abundant non-covalent bond interactions in GPEs to provide good self-healing and adhesion properties of GPEs [63].

#### Preparation Method of GPE for ECDs

The preparation technology of GPEs for ECDs can be categorized into two types: non-in situ and in situ preparation. The non-in situ preparation process involves the advance preparation of GPEs prior to the assembly of ECDs and subsequent assembling the GPEs into the devices. The non-in situ preparation process involves preparing the GPE in advance before assembling the ECDs, while the in situ preparation process involves the assembly of the GPE precursor concurrently as assembling the ECDs and then triggering in situ gelation of the GPE in the device.

##### Non-in situ Preparation of GPE for ECDs

**Fig. 3:**
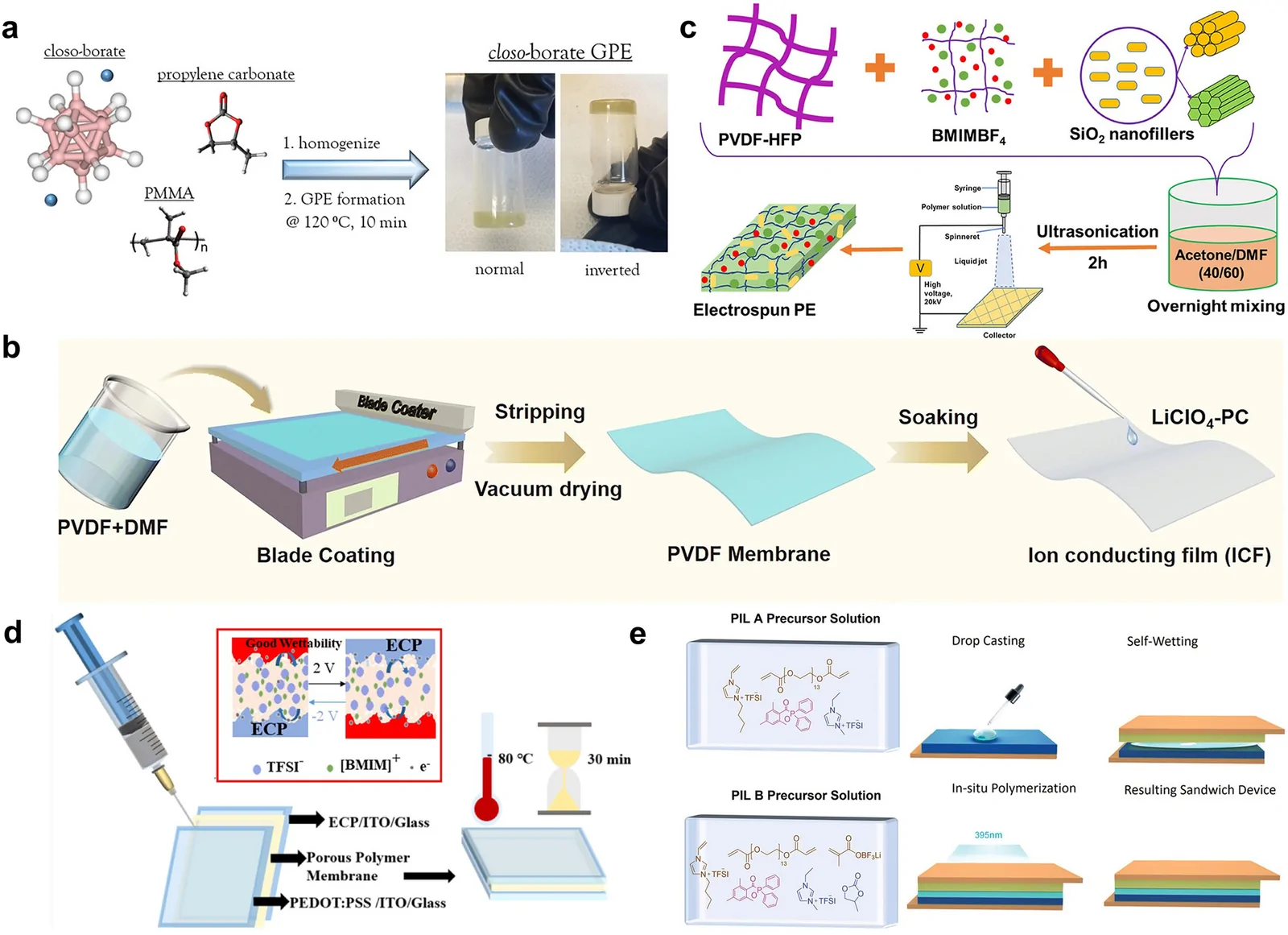
Preparation methods of GPEs. **a** Solution-casting: PMMA was dissolved in PC at high temperature and the solution was poured between the devices, and finally GPEs were obtained after cooling. Reproduced with permission [83]. Copyright 2022, Wiley–VCH Verlag. **b** Phase inversion: The PVDF was first dissolved in DMF and scraped to form a film. Afterward, the DMF is removed by vacuum drying, and the PVDF film was obtained. Finally, the membrane was immersed into the electrolyte and dissolved by the electrolyte to obtain GPEs. Reproduced with permission [84]. Copyright 2023, Royal Society of Chemistry. **c** Electrospinning. Reproduced with permission: The polymer was dissolved in DMF to prepare a polymer solution and made into GPEs by electrostatic spinning. Reproduced with permission [43]. Copyright 2023, American Chemical Society. **d** In situ thermal polymerization: GPEs precursors were infused into ECDs and the polymerization of monomers was initiated by heating to form GPEs. Reproduced with permission [86]. Copyright 2022, Elsevier. **e** In situ UV-light polymerization: GPEs precursors were infused into ECDs and the polymerization of monomers was initiated by UV light to form GPEs. Reproduced with permission [42]. Copyright 2022, Wiley–VCH Verlag

Since the preparation of the gel and the assembly of ECDs are not synchronous, the non-in situ method possesses the advantages of flexible preparation, facile regulation of gel's microstructure, pore distribution, and also holds the potential for large-scale industrial production. The preparation strategies of ex situ GPEs mainly comprise solution-casting, phase inversion and electrospinning methods. Patrick A. Ward et al. prepared the GPE with remarkable electrochemical stability and wide operating temperature window by solution-casting method [83]. As depicted in Fig. 3a, the PMMA was initially dissolved at 120 °C in Li closo-borate-PC solution to form a homogeneous liquid solution. After it cooled down, the GPE was loaded into the ECDs by means of coating. The solution-casting method is straightforward and efficient, but it results in GPEs with poor mechanical strength. Wang et al. have prepared the PVDF-based gel GPE membranes for flexible ECDs by phase transition method [84]. As shown in Fig. 3b, to prepare transparent and ultrathin GPE membranes, researchers first dissolved the PVDF particles with DMF to prepare homogeneous PVDF membranes and then converted DMF to LiClO_4_-PC to achieve high ionic conductivity. In order to finely regulate the structure of GPEs and further control their ion-transport ability, electrospinning technology has been introduced. Through electrospinning technology, the fiber diameter, porosity and structure of GPEs are precisely controlled, thereby improving the overall performance of GPEs [85]. As shown in Fig. 3c, He et al. prepared a GPE for ECDs with high stability by electrostatic spinning process [43]. Due to its uniform structure and nanofillers, GPEs exhibit excellent ionic conductivity and mechanical strength, addressing issues such as high filler loading requirements, low ionic conductivity, sluggish response and poor switching stability in GPE-based ECDs.

##### In situ Preparation of GPEs for ECDs

The solid GPEs fabricated by the non-in situ process fail to fully penetrate into the pores of the electrode material, resulting in poor interfacial contact and high interfacial impedance, which adversely affects the performance of the ECDs [45]. Fortunately, in situ process GPEs were proposed to address the above challenges and simplify the preparation process. In situ GPEs are prepared by in situ physical gelation of polymers, in situ chemical cross-linking of polymers and in situ polymerization of monomers [87]. The precursor for the GPEs should maintain a relatively low viscosity to facilitate effective wetting of the electrochromic functional materials and ensure the optimal contact and interaction between them and the electrolyte. Because polymer chains tend to elevate the viscosity of the gel prepolymer, initiating in situ polymerization of the monomers within the precursor is a more efficient method than performing in situ physical gelation and cross-linking of the polymer. In this approach, a precursor comprising a curable monomer, a liquid electrolyte and an initiator is introduced directly into ECDs and subsequently cured under specific conditions—such as ultraviolet (UV) light, thermal exposure, or electron beam radiation—resulting in the formation of a polymer network, while the liquid electrolyte uniformly filled within the interstices of this network [88, 89].

Thermal initiation, being one of the most prevalently employed methods for initiating monomer polymerization, is frequently utilized to trigger the in situ curing of GPEs. Kuo-Chuan Ho et al. reported an in situ heat-cured GPE based on methyl methacrylate [90]. The resulted thermal-cured GPEs offers remarkably high transparency and superior ionic conductivity, rendering it an ideal electrolyte for ECDs. As shown in Fig. 3d, Zhang et al. injected the GPE precursor into the device and heated it to 80 °C for induce polymerization and form the GPEs [86]. The resultant ECDs present excellent electrochromic performance such as high transmittance contrast, fast switching response and high color efficiency. Compared to thermal initiation, which requires high temperatures and long reaction times (which can be days), UV-initiated polymerization is more efficient and therefore widely welcomed [42, 91]. The curing of GPEs induced by UV light can often be completed in minutes or even seconds, and it also avoids energy waste caused by prolonged heating during thermal curing. As presented in Fig. 3e, Pooi See Lee et al. have prepared poly(ionic liquid) ionogels for flexible and thermally stable ECDs by in situ rapid photopolymerization [42]. The preparation process of in situ rapid polymerization of the ionogels and the good spreading property of its precursor make tight contact between it and electrochromic materials or electrodes, making ECDs have excellent electrochromic performance.

In this chapter, we conduct a detailed review of the fundamental aspects regarding GPEs serving as the electrolyte layer in ECDs. Initially, we comprehensively summarize the performance requirements of ECDs for GPEs, with the aim of offering guidance for the research and development of GPEs. Subsequently, according to the types of plasticizers within GPEs, they are classified into hydrogel electrolytes, organogel electrolytes and ionogel electrolytes. We then review the properties and characteristics of each category separately. Furthermore, based on the types of interactions among polymers, we delve into the gelation mechanism of GPEs. On this foundation, we also present an in-depth review of the preparation methods of GPEs.

## Multifunctionalized GPEs for ECDs

In the practical applications of ECDs, taking into account only the basic properties of electrolytes when designing GPEs has substantial limitations. Analogous to other electrochemical devices, GPEs in ECDs are pivotal in ion transport and improving the safety performance of the electrolyte layer. However, as an intelligent optical modulation apparatus with diverse application scenarios, the performance metrics of ECDs extend beyond mere electrochemical performance. Consequently, as a key component of ECDs, GPEs should also contemplate how to align with the functional requirements of contemporary advanced ECDs, rather than being merely constrained by the fundamental electrochemical and physical characteristics of electrolytes. Therefore, while preserving their outstanding ion-transport capabilities, it is highly imperative to further functionalize GPEs in accordance with the requirements of current advanced ECDs. In this section, we will chiefly review the GPEs that have been functionalized to meet the requirements of ECDs, covering intrinsic electrochromic GPEs, temperature-responsive GPEs, photo-responsive GPEs, as well as self-healing and stretchable GPEs.

### GPEs with Electrochromic Function

Some electrochromic materials, such as viologens, can be compounded into the GPE, thus allowing the gel to behave as both electrolyte layer and electrochromic layer [92–96]. In ECDs, the multilayer structure of ECDs causes serious problems such as complex fabrication, low transmittance and slow response [97]. In particular, frequent-ion insertion and extraction driven by external electric field during coloring and bleaching processes usually severely damage the multilayer structure and degrade the performance [98]. Electrochromic GPEs simplify the device structure by integrating the electrochromic material with the electrolyte layer, effectively addressing the previously mentioned challenges.

**Fig. 4:**
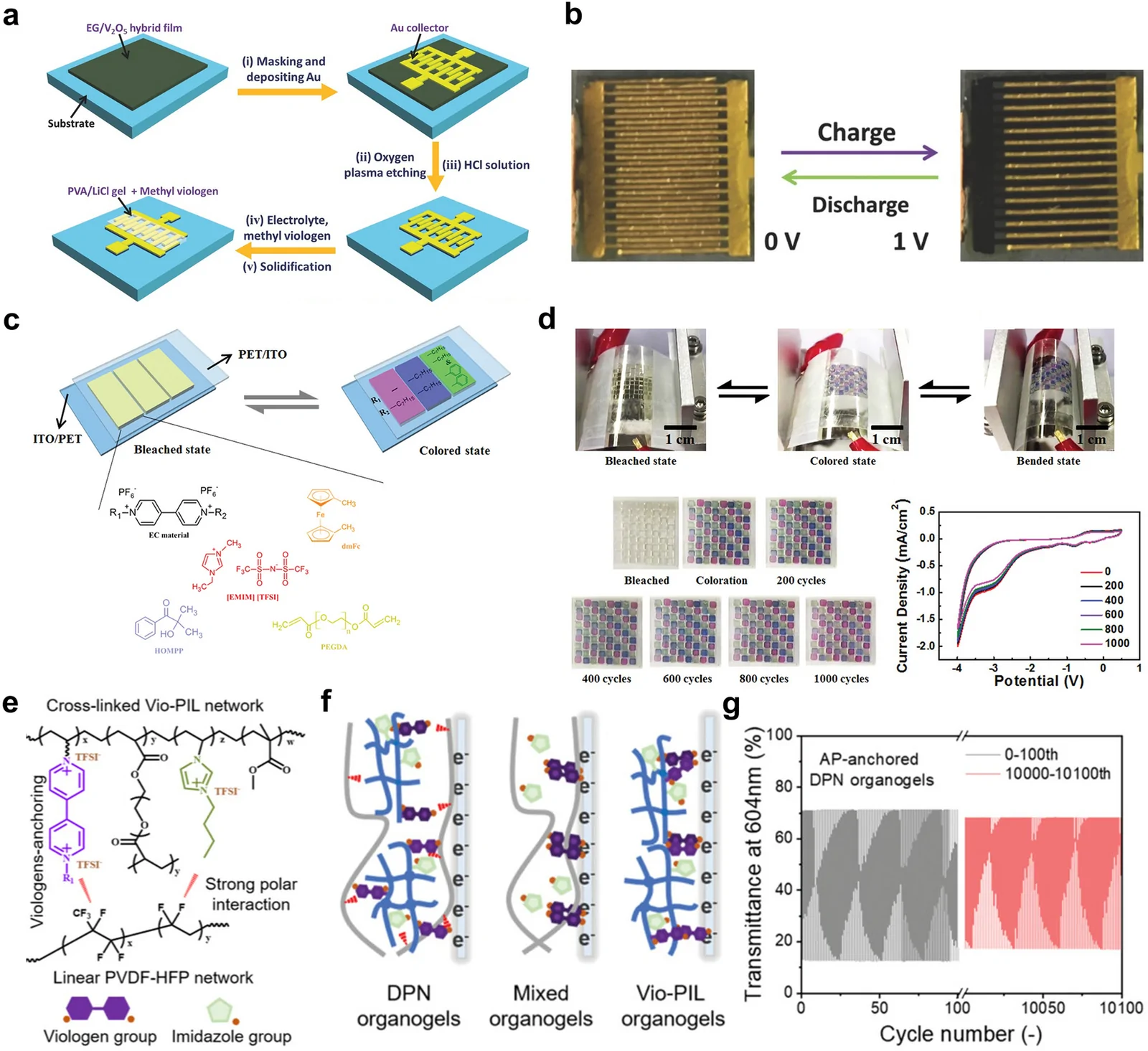
Electrochromic GPEs for ECDs. **a** Preparation process of micro-supercapacitors. **b** Electrochromic properties of the device (charge coloring, discharge fading). Reproduced with permission [104]. Copyright 2017, Wiley–VCH Verlag. **c** Composition and structure of multicolor electrochromic GPEs. **d** Electrochromic performance and stability of the ECDs. Reproduced with permission [105]. Copyright 2019, Wiley–VCH Verlag. **e** Schematic illustration of semi-interpenetrating double network GPEs. **f** Mechanism for cycling stability of the ECDs. **g** Long-term cyclic test results for the ECDs. Reproduced with permission [106]. Copyright 2023, Wiley–VCH Verlag

Different from inorganic electrochromic materials such as WO_3_ and NiO_2_, which are not well soluble in electrolytes, viologens with good solubility in applicable solutions are ideal to make electrochromic GPEs for simplified ECDs [99, 100]. 4,4′-Bipyridinium salts, commonly called viologens (V^2+^), are a well-acknowledged class of electrochromic materials with three reversible redox states, namely V^2+^ (dication, pale yellow colored/colorless) ↔ V˙ (radical cation, viologen/blue/green) ↔ V^+0^ (neutral, colorless) [101]. The electrochromic properties of these materials can be modulated by varying the nitrogen substituents on the pyridyl ‘N’; also, besides this, varying the counter ions with specific functionalities has been shown to enhance the electrochromic behavior, such as switching time, cycling stability and device performance [34, 102, 103]. Feng et al. have prepared an electrochromic GPE by dissolving methyl viologen in polyvinyl alcohol (PVA)/LiCl electrolyte and subsequently solidifying the mixture [104]. As shown in Fig. 4a, the GPEs precursor heated to 85 °C was cast onto the electrode surface and cured overnight to fully cured. As presented in Fig. 4b, the GPEs provide electrochemical functionality for micro-supercapacitors while providing direct visual observation of the capacitor's charging and discharging states through a significant reversible electrochromic effect during charging and discharging. Unlike most electrochromic materials with single color change, viologens can show multiple colors by changing the form and chain length of substituents. Therefore, viologens with different substituents is frequently utilized to prepare multicolor electrochromic displays. As presented in Fig. 4c, Jae-Min Myoung et al. have synthesized IL-based electrochromic GPEs through composite monoheptyl-viologen (MHV), diheptyl-viologen (DHV) and diphenyl-viologen (DPV) to obtain ECDs with colors of magenta, blue and green, respectively [105]. The researchers achieved subpixelated flexible ECDs by sequential UV-curable patterning of magenta, blue and green electrochromic GPEs (Fig. 4d). Consequentially, this work is the first to demonstrate flexible and transparent ECDs with simultaneously implementable subpixelated electrochromic gels as active-matrix display application materials.

Although viologens GPE-based ECDs have the advantages of color variety, rapid response and simple structure, the irreversible aggregation of viologen radicals (also referred to as dimerization) significantly affects the cycle life and utilization efficiency of the ECDs [101]. Fortunately, the polymer backbone in the GPEs can interact with the viologen after rational design, thus inhibiting the dimerization of viologen radicals. Deng et al. firstly synthesized boronic acid viologen (BBV) and subsequently inhibited the dimerization of BBV radicals through the boronic ester linkages between PVA and boronic acid group in BBV in the GPEs [107]. The ECD that based on this intrinsic electrochromic GPE still has high electrochromic performance after 10,000 cycles, which is expected to further promote the application of viologen GPEs. In another research, Wang et al. avoided the dimerization of viologen by anchoring it to polyionic liquid polymer chains with covalent bonds [106]. As shown in Fig. 4e, f, the viologen (1-allyl-1 “-propyl-viologen (AP), 1-allyl-1”-heptyl-viologen (AH) and 1-enheptyl-viologen (ME)) were directly anchoring into the polymer network and further anchored by the electrostatic action of the PVDF. Owing to the fact that this semi-interpenetrating network GPEs inhibited the aggregation of viologen, the ECDs showed excellent cycling stability (Fig. 4g).

### Temperature-Responsive GPEs for ECDs

Temperature-responsive GPEs endow ECDs with thermochromic function and prevent thermal runaway. Thermochromic GPEs are highly attractive for electrochromic smart windows, as they enable dual stimuli regulation by both electricity and heat [35, 36]. For electrochromic energy storage devices, thermo-responsive GPEs that prevent thermal runaway significantly improve the safety of the device [108, 109]. The majority of the thermo-responsive property of GPEs is realized through the reversible phase transition generated at low critical intercalation temperature (LCST) [110, 111]. At temperatures below LCST, there are strong hydrogen bonds between the polymer chains and plasticizers in thermo-responsive GPEs. At temperatures higher than LCST, the hydrogen bonding between the polymer chains and the plasticizer is broken leading to aggregation of the polymer chains and thus causing phase separation within the gel.

**Fig. 5:**
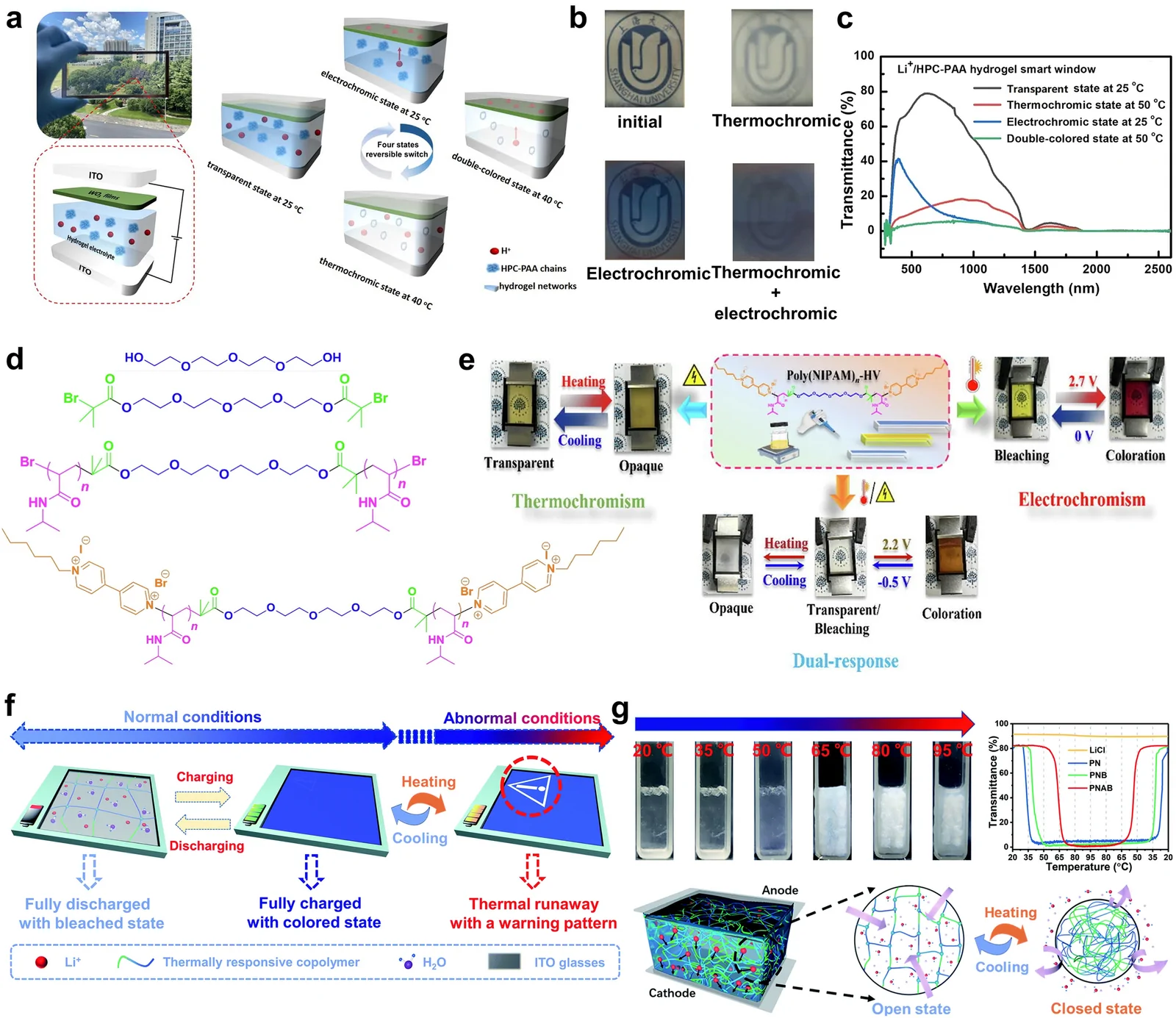
Temperature-responsive GPEs for ECDs. **a** Device structure and four operating modes of ECDs with HPC-based GPEs. **b**, **c** Four color-changing effects of ECDs. Reproduced with permission [117]. Copyright 2023, Elsevier. **d** Synthesis of the poly(NIPAM)_n_-HV. **e** Multi-color-changing effect of the poly(NIPAM)_n_-HV ECDs. Reproduced with permission [118]. Copyright 2024, Elsevier. **f** Working diagram of PNAB-based ECDs. **g** Thermochromic effect and thermal control mechanism of thermal-responsive GPEs. Reproduced with permission [119]. Copyright 2022, Royal Society of Chemistry

Thermochromic gels based on phase transition permit incident light to pass through below the LCST, while strongly scattering incident light at temperatures higher than the LCST due to the scattering center created by phase separation [112, 113]. Recent years, thermochromic gels have been widely used in smart windows [114], sensors [115] and tissue engineering [116] due to their excellent properties such as high transparency at temperatures below LCST, low phase transition temperature and ease of processing. Thermochromic gel can be used as electrolyte to superimpose thermochromic function on ECDs, thus preparing smart devices with both electrical and thermal response. Poly(N-isopropylacrylamide) (poly(NIPAM)n) and hydroxypropyl cellulose (HPC) are two commonly adopted polymers for the fabrication of thermochromic GPEs. Gao et al. have reported an HPC-based GPE with adjustable LCST that can be modulated by ionic species and concentrations [117]. As presented in Fig. 5a, the hydrogel undergoes a thermochromic transition from a highly transparent state to a milky white state and can be used as a GPE for WO_3_-based electrochromic devices, resulting in electrically and thermally dual-responsive devices. As presented in Fig. 5b, c, this dual-responsive device can reversibly and rapidly switch among four colors of highly transparent state (bleached state), milky white state (thermochromism), blue color state (electrochromism) and double-colored fully nontransparent state (electric-/thermal-dual-responsive state) by regulating the voltage and temperature. In the previous section, we provided a detailed discussion on GPEs that inherently possess electrochromic properties. As a result, both electrochromism and thermochromism can be simultaneously integrated into GPEs, eliminating the need for an additional electrochromic layer and simplifying the device architecture. Hern Kim et al. have fabricated a poly(NIPAM)_n_-HV-based GPEs with both thermochromic and electrochromic functions by anchoring active viologen into the thermal response poly(NIPAM)_n_ (Fig. 5d) [118]. As can be seen from Fig. 5e, the device utilizing the GPE not only enables independent thermochromic and electrochromic discoloration, but also facilitates simultaneous thermoelectric dual-response discoloration. The device has almost zero transmittance in both visible and infrared light after undergoing thermochromic and electrochromic changes, which has great potential for applications.

Heat accumulation resulting from the rapid migration of charge/ions during charging/discharge may trigger exothermic chemical reactions within the energy storage device, eventually leading to serious safety problems such as explosions or fires [120]. Hence, corresponding measures to prevent thermal runaway are urgently required to enhance the safety of the device without compromising its performance. At elevated temperatures, poly(NIPAM)_n_ transitions from a hydrophilic to a hydrophobic state as hydrogen bonds between the N-isopropyl groups and water are broken, resulting in the formation of hydrogels through hydrophobic interactions that inhibit the movement of conductive ions between the electrodes [121–123]. This process reverses upon cooling, restoring both electrochemical performance and power delivery capacity of the energy storage device. As presented in Fig. 5f, Yan et al. have successfully fabricated intelligent electrochromic supercapacitors with self-thermal runaway protection and in situ real-time monitoring by employing poly(NIPAM)_n_-based thermal response GPEs [119]. To fabricate a thermo-responsive GPEs with appropriate phase transition temperature, N-Isopropyl acrylamide, acrylamide, N, N '- methylene-bisacrylamide were copolymerized (PNAB) to obtain the desired LCST. As shown in Fig. 5g, when the temperature is higher than the LCST, the transparency of the GPE decreases from 80% to nearly 0%. And at the same time, its conductivity also decreases significantly resulting in the system being in a close state. This strategy has great reference value for the safety problems of energy storage equipment caused by thermal runaway.

### Photo-response GPEs for ECDs

Photo-response gel is a novel type of intelligent gel material that exhibits rapid and controllable responsiveness to photo-signal. Owing to their exceptional attributes, such as rapid swift response speed, robust reversibility and precise control, photo-response gels possess immense potential for applications in the fields of smart materials, optical devices and others [124–127]. When combined with ECDs, photo-response GPEs typically consist of a photosensitive substance embedded within a gel matrix, enabling deformation and color change upon illumination. The GPEs with photo-response performance can be used in ECDs to make the devices have electro-optical dual-response characteristics, thus enriching the function of the devices.

**Fig. 6:**
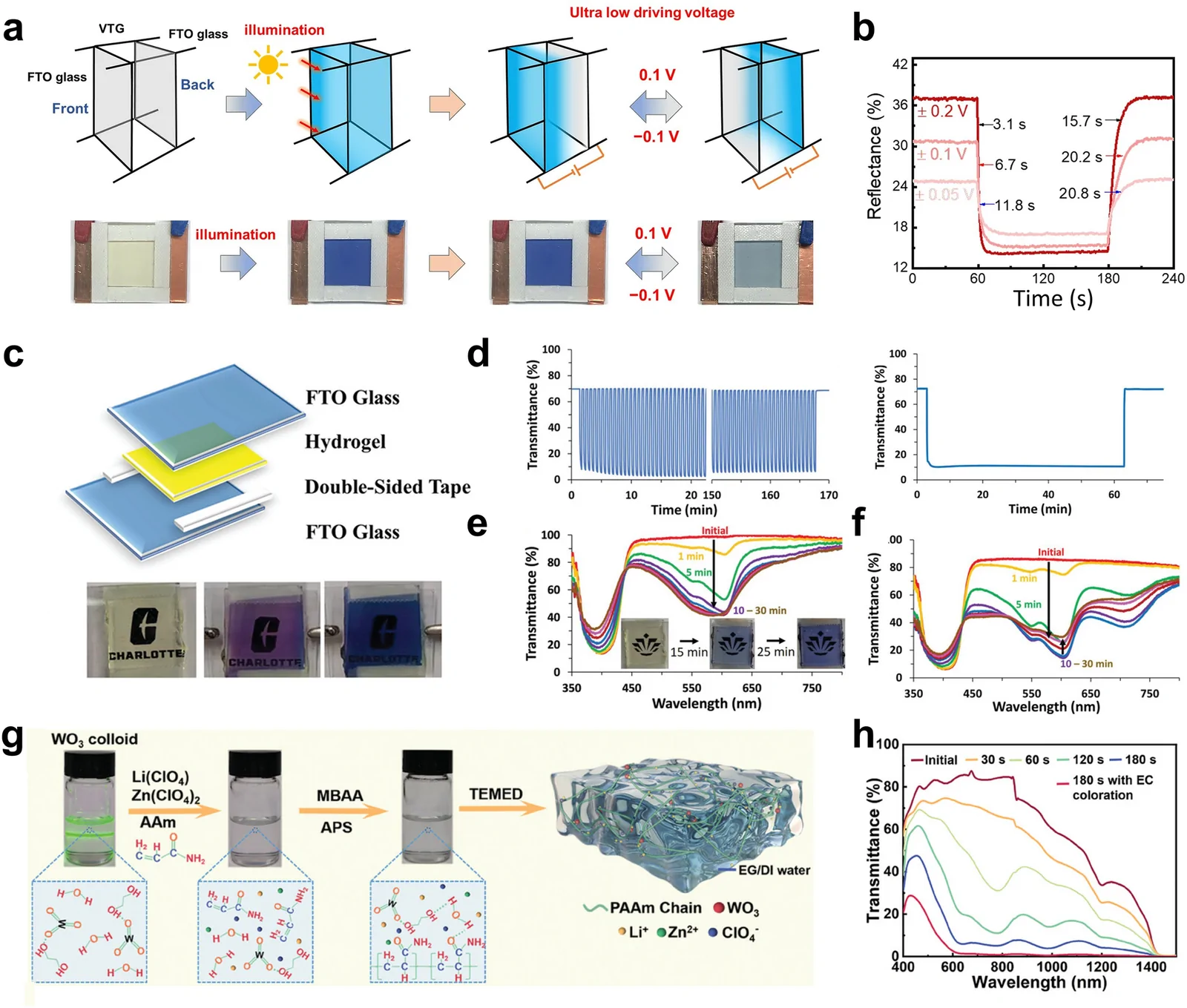
Photo-response GPEs for ECDs. **a** Schematic structure and photo-assisted low-voltage electrochromic performance of ECDs based on viologen/TiO_2_ composite photo-response GPEs. **b** Photo-assisted electrochromic properties of the ECDs. Reproduced with permission [32]. Copyright 2023, Elsevier. **c** Structure and color change of the ECDs based on TTz viologens GPEs. **d** Electrochromic performance of the ECDs. **e** Photochromic performance of the ECDs. **f** Photochromic performance of the ECDs under voltage assistance. Reproduced with permission [24]. Copyright 2021, Wiley–VCH Verlag. **g** Process for the preparation of hydrogel electrolytes based on ethylene glycol (EG)-capped WO_3_ nanodots. **h** Optical transmittance spectra of the ECDs under different durations of sunlight exposure. Reproduced with permission [128]. Copyright 2025, Wiley–VCH Verlag

Wang et al. have prepared a viologen/TiO_2_ composite GPEs with photo-induced self-reduction and coloration, thereby achieving a device that exhibits remarkable electrochromic performance under an ultra-low driving voltage of ± 0.1 V (Fig. 6a, b) [32]. The photochromic properties of the GPEs were enhanced with the increase of TiO_2_ content, light intensity and temperature. Nevertheless, the GPEs still demonstrated excellent photochromic properties even in poor light (i.e., cloudy and rainy days) and over a wide temperature range (–20 and 70 °C). More importantly, the electrochromic response time of the device is significantly shortened when exposed to light and also decreases with the increase of voltage, indicating that in addition to endowing the gel with photochromic functionality, the combination of TiO_2_ also enables the device to rapidly drive its electrochromic change with low voltage under light. In addition, the ECD prepared by this strategy has extremely low energy consumption, and the commercial button battery (1.5 V) simultaneously drives ten ECDs in series and enables color switching, providing new opportunities for fundamental research on the photoelectric and photoelectrochemical properties of emerging functional materials.

The rationally designed viologen essence molecule is expected to act alone as a functional component in GPEs to realize both electrochromic and photo-responsive ECDs. Viologen, as a photochromic material, has its photochromic effect influenced by several factors. The quick antielectron transfer rate can impede this effect, while the presence of oxygen or other oxidants in the system can speed it up. It is expected that proper modification methods to stabilize the photo-induced free radicals of viologen will help to attain photochromic characteristics while preserving its fine electrochromic properties. Based on this consideration, Zhou's group prepared alkyl viologen with thiophene or ethylidene dioxy thiophene bridges to expand the photo-response range of viologen and stabilize the photo-induced free radical intermediates [37]. Subsequently, by using ammonium cation and sulfonate anion as the sealing agents, the synthesized alkyl viologen with a thiophene-derived bridge was completely dissolved in a PAAm hydrogel for the preparation of electrochromic and photochromic GPEs. Although the researchers successfully prepared and tested gel devices with electrochromic and photochromic capabilities, they merely demonstrated the photo- and electro-responses of the devices separately without designing the synergistic effects of these responses. Michael G. Walter et al. incorporated synthetic chromogenic thiazolo(5, 4-D)thiazole (TTz) viologens into PVA/borax hydrogels, thereby generating high-contrast electrochromism, electrofluorochromism and photochromism devices (Fig. 6c) [24]. As shown in Fig. 6d, the device exhibited excellent electrochromic properties, featuring high reversibility, cyclability and durability. In addition, the device also displayed excellent photochromic performance, which could generate significant photochromic effects under light within ten minutes (Fig. 6e) and be maintained for 1–2 h after the light was removed or fade quickly under applied voltage. More importantly, the photochromic performance of the device could be significantly enhanced in the presence of applied voltage, thus further enhancing the color-changing efficiency of the device (Fig. 6f).

WO_3_ has also been reported as a photochromic material. Li et al. prepared photochromic GPEs by compositing ethylene glycol (EG)-capped WO_3_ nanodots in hydrogels (Fig. 6g) [128]. The introduction of EG-capped WO_3_ colloid further enhances the stretchability and self-healing properties of GPEs, in addition to imparting good photochromic properties. In order to achieve excellent performance of photochromic GPEs, the researchers screened the cations in the hydrogels and ultimately selected small-molecule lithium ions for high photochromic performance and zinc ions for zinc anodes. As shown in Fig. 6h, the ECDs based on this photochromic GPEs exhibit excellent optical modulation performance under light irradiation and energization and are expected to be used in dynamic windows and augmented reality (AR) glasses.

### Self-healing and Stretchable GPEs for ECDs

The stretchable self-healing ECDs possess the capability to adapt to various deformations of the substrate and undergo self-recovery after sustaining damage, thereby enhancing their applicability and durability [129, 130]. However, it remains difficult to realize mechanical stretchability and self-healing properties in ECDs, with the primary challenge residing in the identification of optimal electrolyte materials that offer outstanding mechanical stretchability and self-healing capability, in addition to conductivity [82, 131–134]. The diversity of polymer science and polymer engineering provides an extensive repertoire for designing and tailoring GPEs, offering excellent flexibility and adaptability. Therefore, among various electrolyte materials, GPEs with adjustable physicochemical properties enable the realization of stretchable and self-healing ECDs.

**Fig. 7:**
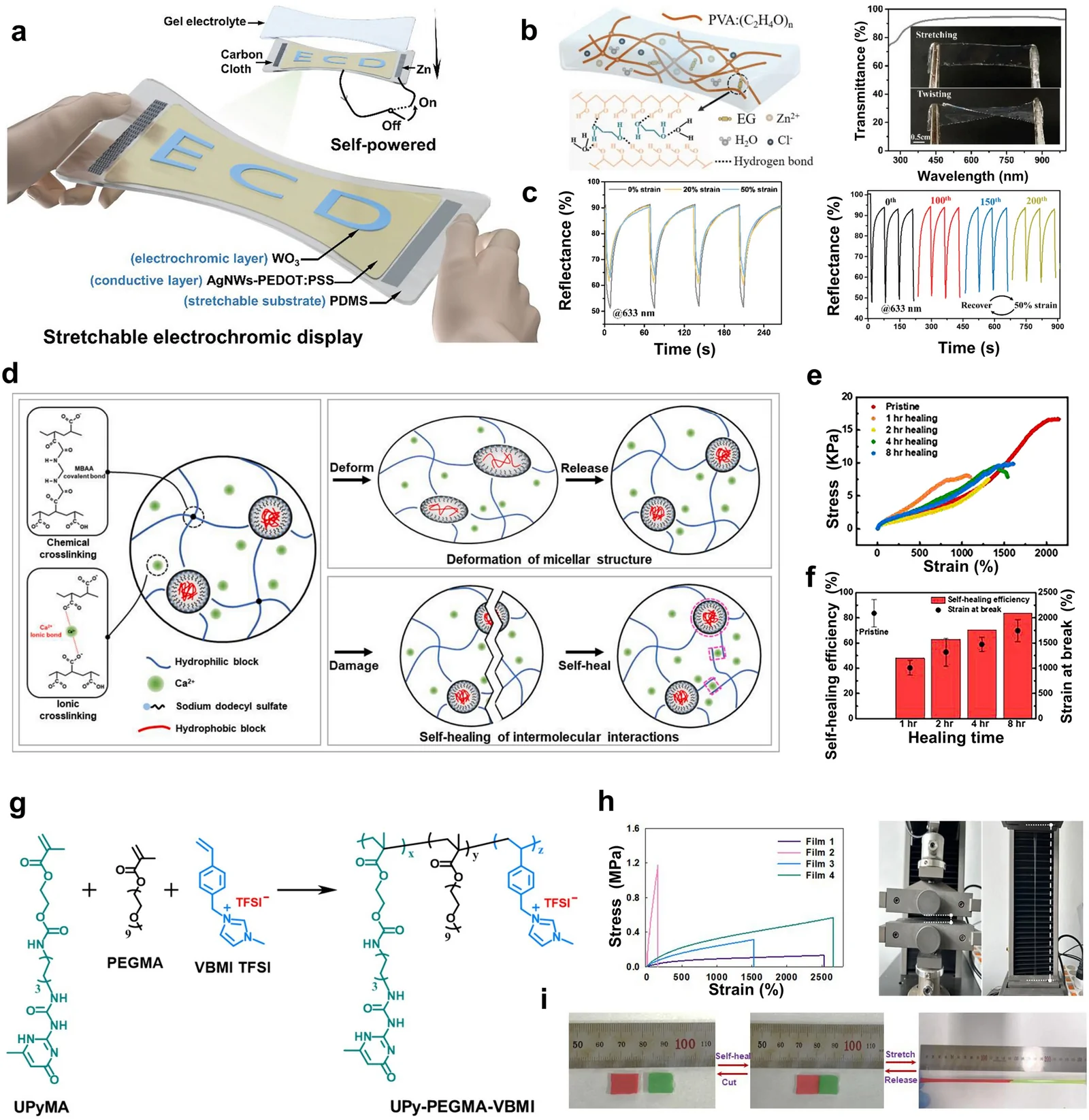
Self-healing and stretchable GPEs for ECDs. **a** Schematic structure of stretchable electrochromic display. **b** Structure and properties of stretchable GPEs. **c** Electrochromic performance of the stretchable electrochromic displays under tension. Reproduced with permission [136]. Copyright 2023, Wiley–VCH Verlag. **d** Illustration of self-healing and stretchable mechanism of the GPEs. **e, f** Self-healing performance of the GPEs. Reproduced with permission [131]. Copyright 2022, Elsevier. **g** Design strategy for self -healing and stretchable GPEs based on quadruple hydrogen bonding interactions. **h** Stretchability of GPEs. **i** Self-healing performance of GPEs. Reproduced with permission [137]. Copyright 2024, Elsevier

Stretchable GPEs have attracted considerable attention in recent research due to their remarkable performance when subjected to large deformations. Through a well-conceived molecular design and meticulous control of the multi-level structure, it is feasible to significantly enhance the stretchability of these GPEs. The GPEs' stretchability is strongly influenced by both the type of cross-linking bond and the density of cross-links. Cross-linking methods that rely on non-covalent interactions and feature a low cross-linking density can reduce the material's mechanical strength but facilitate greater stretchability [135]. As shown in Fig. 7a, Pooi See Lee et al. have fabricated the stretchable electrochromic display by employing a stretchable PVA organic hydrogel electrolyte [136]. The organic hydrogel electrolyte utilizes PVA as the polymer framework and ZnCl_2_ ethylene glycol/H_2_O solution as the plasticizer, thus obtaining mechanical stretching and twisting properties by forming a hydrogen bond between ethylene glycol and PVA chain and inducing PVA crystallization (Fig. 7b). The device composed of the GPE exhibits reversible and stable electrochromic properties at tensile (0–50% strain) and 200 stretch/recovery cycles as illustrated in Fig. 7c, which has great application prospects in future stretchable electronic display devices.

Beyond mechanical stretchability, self-healing properties has gained increasing significance because it is difficult to avoid damage to ECDs during long-term use [25, 138, 139]. Self-healing materials are capable of autonomously recovering their original properties after sustaining damage, without the requirement for external stimuli, thus improving the reliability of devices and prolonging their operational lifespan. The recovery of molecular interactions following the application of external forces or damage is predominantly governed by two main types of bonding: dynamic covalent bonds [138, 140] and dynamic non-covalent bonds [25, 82, 141]. Dynamic chemical bonds encompass reactions such as the Diels–Alder reaction, disulfide bonds, Boronic Ester Bonds, Schiff Base Bonds, while dynamic non-covalent interactions consist of hydrogen bonding, ionic interactions (metal coordination), host–guest complexes, and hydrophobic interactions [135]. Jeong Sook Ha et al. reported the fabrication of a self-healing and electrochromic display of hydrogels based on multiple cross-linked networks composed of hydrophilic and hydrophobic waters [131]. As illustrated in Fig. 7d, the hydrogel electrolyte optimizes its mechanical and self-healing properties through chemical cross-linking based on N,N' -methylene bisacrylamide and ionic cross-linking based on CaCl_2_. Benefiting from micelle-based multiple cross-linked networks, the GPE possesses excellent self-healing properties (recovering to more than 80% of its initial properties after eight hours of self-healing at room temperature), and it did not decrease significantly after ten damage healing cycles (Fig. 7e, f). As previously described, the stretchability and self-healing properties of GPEs are dependent on dynamic covalent or non-covalent bonding interactions that possess reversibility. Thus, the stretchability and self-healing properties of GPEs are expected to be integrated simultaneously. As shown in Fig. 7g, Jong S. Park et al. prepared a ternary copolymer with quadruple hydrogen bonds (UPy-PEGMA-VBMI) to achieve self-healing and stretchability in GPEs [137]. The GPEs exhibit excellent stretchability and self-healing properties (Fig. 7h, i), introducing an innovative approach for the fabrication and novel applications of stretchable large-area flexible devices.

In this section, we provide a detailed summary of GPEs functionalized toward the needs of ECDs, including intrinsically electrochromic, temperature-responsive, photo-responsive, and stretchable self-healing GPEs. The advantages of these four functionalized GPEs, the main functional materials and their corresponding properties are summarized in detail in Table 2. In comparison with conventional ECDs, ECDs based on functionalized GPEs manifest numerous notable advantages, such as diverse response characteristics and stronger durability. Nevertheless, the functional modification of GPEs may also lead to the attenuation of other properties of ECDs, such as response rate and electrochemical stability. Consequently, in the future development of GPEs for ECDs, it is essential not only to place emphasis on their functional design but also to explore strategies for reconciling the other issues that arise from such functional design approaches.

**Table 2:** Advantages, key materials and performance comparison of intrinsic electrochromic GPEs, temperature-responsive GPEs, photo-response GPEs and stretchable self-healing GPEs

Functionalized GPEs	Advantages for ECDs	Key materials	Color change and optical modulation	Response time Coloration/Bleaching	Cyclic stability	Reference
Intrinsic electrochromic	Simplified device structure	Methyl viologen	Colorless- purple	From several minutes to only a few seconds at the current densities from 0.01 to 0.4 mA cm^−2^	200	[104]
		MHV, DHV and DPV	Transparent- emitted magenta, blue and yellowish-green colors 90%	19 s/31, 20 s/34 s and 39 s/21 s	3600 s	[105]
		BBV	Transparent- dark blue 71.33%	3.9 s/7.4 s	10,000， 82.66%	[107]
		AP, AH and ME	62%	29 s/11 s, 27 s/13 s and 22.5 s/14.5	10,000， 87.5%	[106]
Temperature responsive	Enables ECDs to integrate thermochromic and thermo-responsive gating	HPC	Thermochromic: Transparent—milky white 70.3% Electrochromic: Transparent -dark blue 66.1%	7 s/35 s	100； 650	[117]
		Poly(NIPAM)_n_-HV	Transparent-opacity—orange hue 85.13%	0.5 s/3.72 s	1000	[118]
		PNB	Thermochromic: 80% Electrochromic: transparent- deep blue 63.5%	No mentioned	500	[126]
Photo-response	Enabling integrated photochromism in ECDs	Viologen/TiO_2_	Colorless- blue 91.3%	3.1 s/15.7 s	1500	[32]
		Alkyl viologen with thiophene or ethylidene dioxy thiophene bridges	Photochromic: Transparent- light red/green- reddish brown Electrochromic: Colorless- purple/reg/green-dark blue/reddish brown. 68%-78%	Photochromic:t_1/2_ = 39 s- 65 s Electrochromic: 2.8 s-8 s	Photochromic 100 Electrochromic 1000	[37]
		TTz viologens	Photochromic: Bleached-Blue. 50% Electrochromic: Bleached-Purple-Blue. 75%	Photochromic: 10 min Electrochromic: 2 s-12 s/0.8 s-4.8 s	250	[24]
		EG-capped WO_3_	Photochromic: Bleached-Blue 90.2 Electrochromic: 57.7%	30 s/5 h 4.6 s/1.3 s	1000	[128]
Stretchable and self-healing	Improve the durability and the wearability of ECDs	ZnCl_2_-EG/H_2_O-PVA organohydrogel	40% (0 strain), 30% (20%strain), 27% (50% strain)	2.5 s (0% strain), 4 s (20% strain), 4.7 s (50% strain) /35 s-36 s	200; tensile 400	[136]
		N,N' -methylene bisacrylamide and CaCl_2_	Transparent- deep purple 76.1%	15 s/within 20 s	90	[131]
		UPy-PEGMA-VBMI	Transparent- deep purple 81.3% (0% strain) 42.7% (53% strain)	5.3 s/15.1 s	100; tensile 300	[137]

## Application of GPEs in ECDs

With the growing demand for intelligence and sustainability, ECDs are increasingly being applied in various fields, such as smart windows, energy storage devices, displays and wearable electronics. GPEs integrate the merits of both solid and liquid electrolytes, affording high ionic conductivity while avoiding device failure due to electrolyte leakage. Moreover, GPEs with additional functionalities can further enhance the performance of ECDs or diversify their features. Based on the above background, this chapter will delve into the applications of the GPE-based ECDs such as smart windows, energy storage devices, displays and wearable electronics, with a particular focus on their pivotal role in enhancing the performance of ECDs.

### Application of GPEs in Electrochromic Smart Windows

Currently, a significant amount of energy is used for heating, ventilation and air-conditioning to maintain comfortable indoor temperatures [142, 143]. As the energy demand for building cooling and heating continues to ascend, reducing the energy consumption of these building services while maintaining a high level of comfort inside these building is critical for achieving sustainable development. To address this issue, smart window technology has emerged, being capable of regulating the amount of sunlight transmitted while reducing the energy consumption associated with ventilation, heating and air-conditioning systems [6, 144]. The effectiveness of daylighting in buildings relies heavily on the visible-light spectrum (VIS, 400–760 nm), and the remaining 50% of solar radiation that utilized for interior heating purposes is primarily located in the near-infrared region (NIR, 760–2500 nm) [145, 146]. In the visible-light modulation category, the transparency can be adjusted as needed to realize the privacy protection function. When privacy is needed, the window is darkened or made opaque; when light is needed, transparency is restored. The ability to modulate the NIR transmission through the windows has a significant effect on regulating indoor living temperature and reducing energy consumption. It is worth noting that a mere 1 °C change in indoor temperature can result in a 10% reduction in energy consumption [147, 148].

**Fig. 8:**
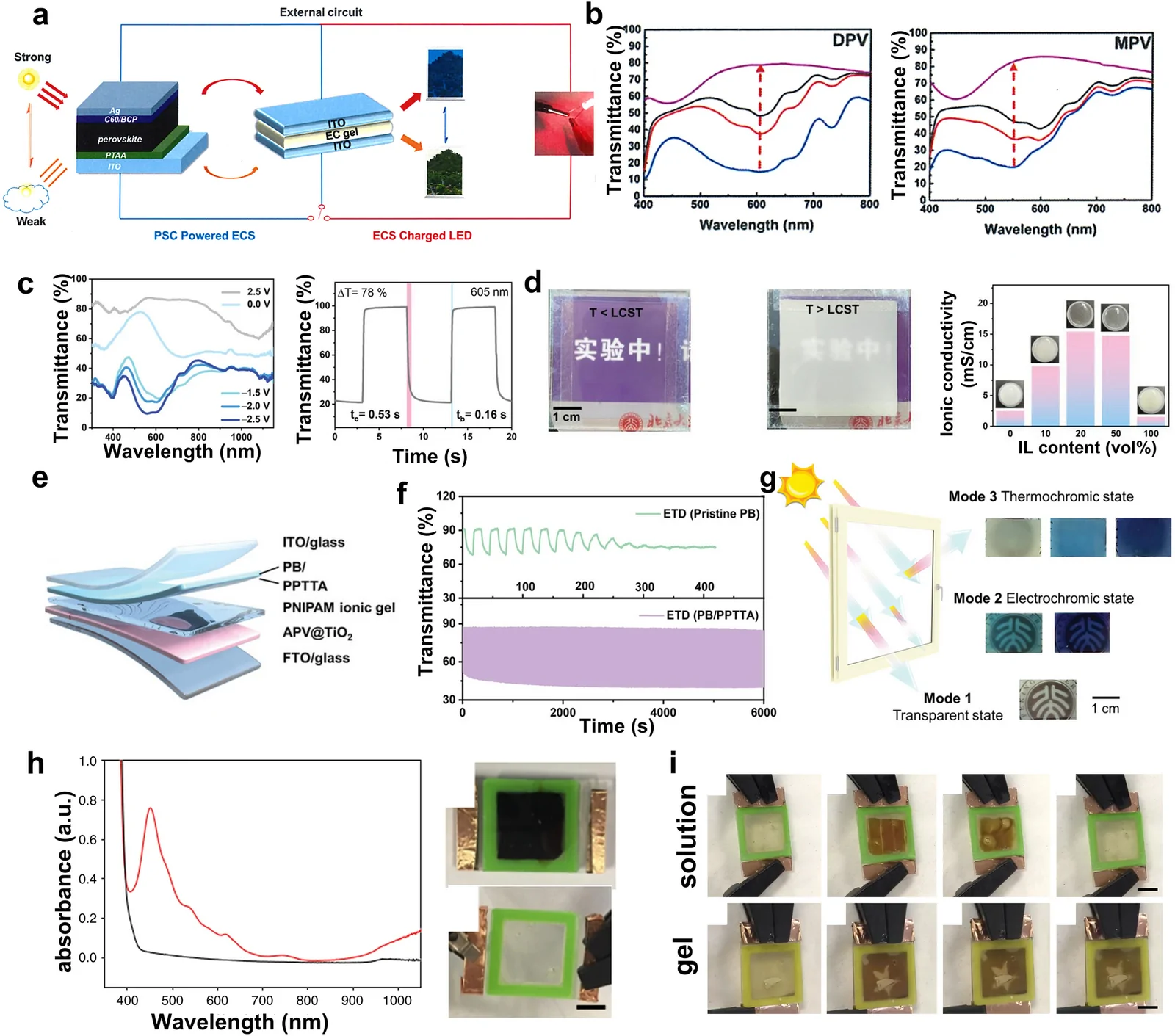
Application of GPEs in electrochromic smart windows. **a** Structure and working principle of the PSC-based electrochromic smart window. **b** Electrochromic performance of the device with compositing different viologen. Reproduced with permission [152]. Copyright 2021, Springer Nature. **c** Electrochromic performance of APV-based ECDs.** d** Thermochromic properties and ionic conductivity of the GPEs. **e** Structure of the electrochromic and thermochromic smart windows. **f** Electrochromic cycling stability of the smart windows. **g** Multiple operating modes and color-changing effects of the smart windows. Reproduced with permission [35]. Copyright 2023, Wiley–VCH Verlag. **h** Dual stimuli-responsive behavior of smart windows based on photochromic and electrochromic GPEs (UV light coloring shown in red; voltage bleaching in black).** i** Patterned solutions and gel devices. Reproduced with permission [153]. Copyright 2018, Springer Nature

Electrochromic windows can reversibly control the transmittance of solar radiation by applying different voltages, offering advantages such as high optical modulation amplitude, long lifespan and active control [7, 149, 150]. Yong et al. prepared PMMA-based GPEs by UV irradiation polymerization and applied them in electrochromic smart windows [151]. Thanks to the excellent performance of GPEs, ECDs exhibit excellent optical modulation, fast response and environmental stability over a wide temperature range. ECDs based on the GPEs have been proved can effectively monitor sunlight and UV exposure in real smart window applications and have excellent potential for practical applications. Current electrochromic smart windows still rely on an external power source and have difficulty adjusting the external light autonomously. As shown in Fig. 8a, Liu et al. developed an all-in-one ECD based on viologen gel and seamlessly integrated it with a perovskite solar cell (PSC), resulting in the creation of an intelligent electrochromic window capable of automatically regulating solar radiation [152]. In order to prepare electrochromic windows with long-term stability and multi-color intelligently adjustable electrochromic windows, the researchers first successfully synthesized monoalkynyl-substituted viologen and dialkynyl-substituted viologen, respectively, and then combined them into the gel. As shown in Fig. 8b, driven by PSC, the transmittance of the two ECDs in the visible range gradually increases with the decrease of optical density. The PSC-driven viologen gel-based ECDs strategy has great application prospects in the field of intelligent windows.

In addition to ECDs, thermochromic devices are also widely employed in smart windows [154, 155]. Compared with ECDs, thermochromic smart windows can dynamically adapt to the changes of solar and thermal radiation without the assistance of external voltage and other equipment, which has attracted extensive attention [114]. The single color and passive temperature response characteristics of thermochromism pose challenges in meeting individual aesthetic preferences and privacy requirements, despite its promising application prospects in the field of smart windows. Therefore, integrating thermochromic properties into electrochromic smart windows enables both active optical modulation to meet personalized needs and adaptive optical modulation for dynamic responsiveness [117, 145, 156]. As mentioned above, with appropriate design, the GPEs used in ECDs can exhibit thermochromic properties that meet the requirements of smart windows, enabling the preparation of double responding intelligent windows efficiently. Hern Kim et al. have successfully synthesized by facile incorporation of a modification on-symmetric viologen onto the triazole moiety of a phase-changing poly(NIPAmn-TEG) via quaternization to produce dual-responsive poly(NIPAmn-TEG-BPV) [157]. Thermochromic, electrochromic and electrolyte functions are concentrated in the poly(NIPAmn-TEG-BPV)-based gel, thus simplifying the structure of the device. Although the incorporation of viologen into GPEs for the preparation of electrochromic smart windows has shown good feasibility, there are still issues with slow color-changing speed and uneven coloring film [158, 159]. In order to improve the electrochromic response speed and homogeneity of smart Windows, Meng's team adopted a strategy of anchoring electrochromic molecules (viologen) directly to the electrode material [35]. A unilateral phosphonic acid (anchoring group) design was used to prepare viologen-based ECDs with excellent color switching speed and high coloring efficiency and, while retaining the unilateral anchoring design, replaced benzonitrile with allyl to obtain a more transparent material (APV). As shown in Fig. 8c, the ECDs based on the APV exhibit excellent electrochromic performance and extremely fast response speed. Subsequently, ionic gel with high ionic conductivity and thermochromic properties (Fig. 8d) were used as GPEs of the device to achieve more energy-efficient building systems. Finally, the electrochromic smart window was prepared according to the structure shown in Fig. 8e and exhibited excellent cycling stability (Fig. 8f). As presented in Fig. 8g, the smart window showed three modes and six color changes, establishing a promising path for the industrialization of energy-saving smart windows.

In addition to thermochromism, the combination of photochromism and electrochromism holds great promise in the domain of smart windows. Emily R. Drape et al. prepared a GPEs with both photochromic and electrochromic properties by the self-assembly of a naphthalene diimide [153]. The researchers constructed a sandwich ECD with FTO and used in situ polymerization to form a gel in the ECD. As presented in Fig. 8h, when exposed to sunlight the naphthalene diimide-based device will darken without an applied current and bleach under a low voltage. In addition, GPEs have been proved to have better stability, thus showing clear and stable patterned performance (Fig. 8i). The strategy of combining photostimulation and electrochemical stimulation offers significant advantages and presents an intriguing and potentially valuable alternative for smart windows. Through rational molecular structure design, viologen is expected to possess both electrochromic and photochromic properties. Liu et al. successfully synthesized extended violet essence derivatives with both photochromic and electrochromic properties and subsequently dissolved them in GPEs [160]. ECDs based on the GPEs exhibit good photochromic performance and can be maintained for several hours after removal of the light source. And the spectral response of the device extends into the NIR region due to the extended length of the coupler of the chromophore compared to conventional viologen based devices. In conclusion, the ECDs with good electrochromic performance and photochromic performance can realize four working modes, which has a strong application potential in the field of smart windows.

### Application of GPEs in Electrochromic Energy Storage Devices

Electrochromic energy storage devices, which integrate both electrochromic and energy storage functions within a single device, have become a research hotspot in the field of electrochromism [161–163]. In electrochromic energy storage devices, the energy storage level is directly indicated by color changes, enabling visual monitoring of the charge status [164, 165]. Additionally, electrochromic energy storage devices are expected to alleviate the reliance of traditional ECDs on external power supplies, thereby facilitating the application of ECDs [166]. Extensive research has been conducted on the utilization of GPEs in energy storage devices. Due to their high ionic conductivity, good stability, close contact with electrode materials, dendrite suppression and ion-transport regulation, GPEs have become one of the most commonly used electrolyte materials in energy storage devices [47, 167, 168].

**Fig. 9:**
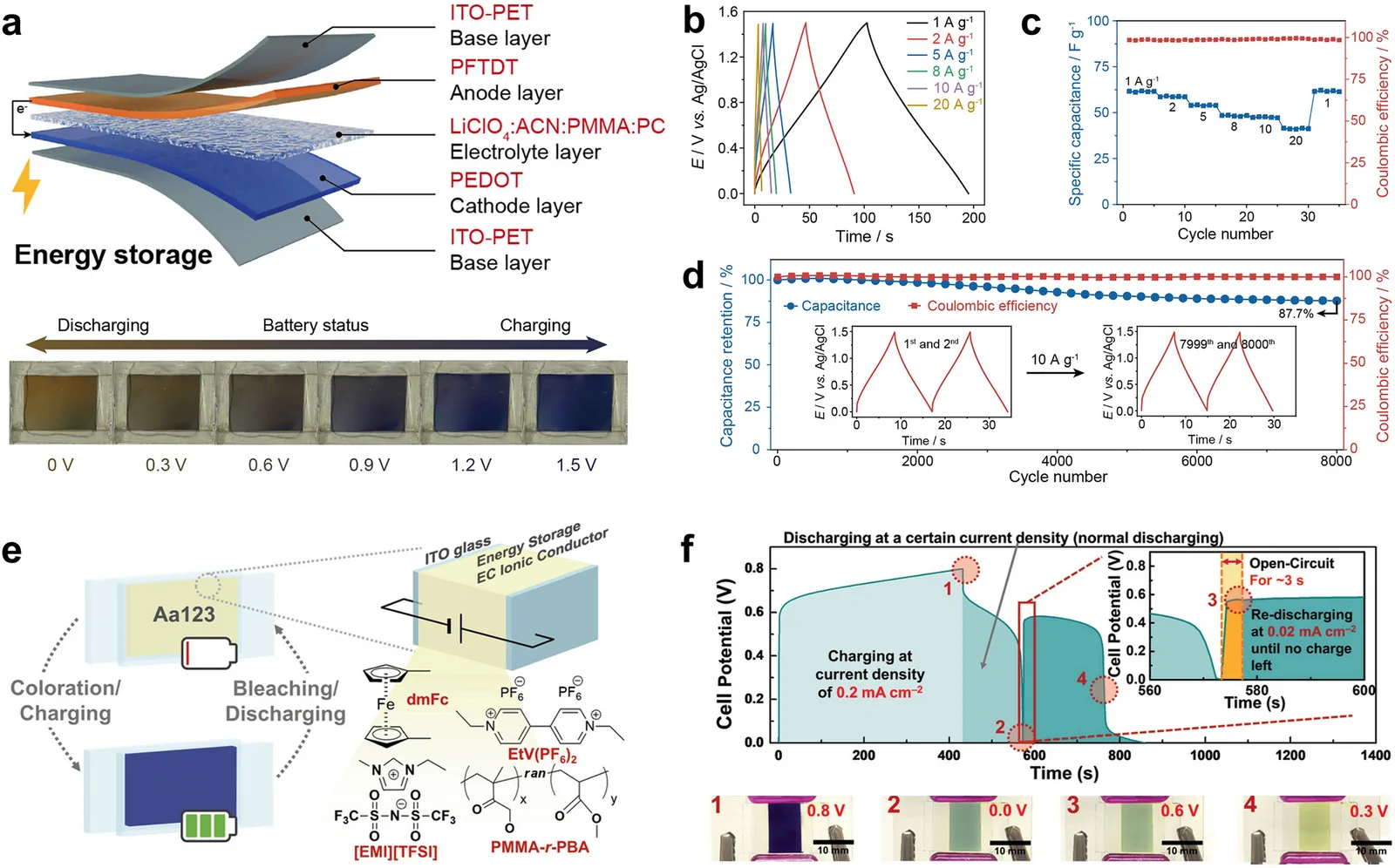
Application of GPEs in electrochromic energy storage supercapacitors. **a** Structure of electrochromic supercapacitors for camouflage and the electrochromic effect of its charging and discharging. **b** GCD curves of electrochromic supercapacitors for camouflage. **c** Coulombic efficiency and specific capacitance of electrochromic supercapacitors for camouflage. **d** Cyclic stability of electrochromic supercapacitors for camouflage. Reproduced with permission [171]. Copyright 2025, Elsevier. **e** Working principle and structure of electrochromic supercapacitors with tailoring diffusion dynamics. **f** GCD curves of electrochromic supercapacitors, accompanied by images showing electrochromic effects at different stages. Reproduced with permission [172] Copyright 2022, Wiley–VCH Verlag

Supercapacitor is a novel type of efficient energy storage devices that have garnered significant attention due to their high power density, long cycle life and rapid charging and discharging capabilities [169, 170]. GPE-based electrochromic supercapacitors, featuring real-time and intuitive display of the stored energy state, have been successfully developed and exhibit promising application prospects. As illustrated in Fig. 9a, Nie et al. prepared electrochromic supercapacitors for camouflage by using organogel as the electrolyte [171]. The GCD curves exhibit symmetrical quasi-triangular shapes across a broad current density range (1–20 A g^−1^), indicative of highly reversible charge storage behavior and robust electrochemical kinetic properties (Fig. 9b). The device demonstrates excellent charging/discharging multiplicity performance, achieving 62 F g^−1^ at 1 A g^−1^ with 62.9% capacitance retention (39 F g^−1^) at 20 A g^−1^ (Fig. 9c). In addition to this, the device has excellent cycling stability, with almost no degradation after 8000 cycles (Fig. 9d), suggesting that it has great practical value. Hong Chul Moon et al. developed a single-layer supercapacitor utilizing an electrochromic ionogel and demonstrated the effect of tailored diffusion kinetics on the energy storage capabilities of the supercapacitor [172]. As illustrated in Fig. 9e, the electrochromic ionic GPEs consist of poly(methyl methacrylate-ran-butyl acrylate) as a polymer framework, [EMI][TFSI] as a plasticizer and ethyl viologen hexafluorophosphate EtV(PF_6_)_2_ (denoted as EtV^2+^) and 1,1'-dimethylferrocene (dmFc) as cathodic electrochromic material and counter anodic materials, respectively. This supercapacitor, utilizing the electrochromic ionic gel, not only demonstrated its in situ energy storage capacity through alterations in light transmittance (or color intensity) but also enhanced both energy storage and electrochromic efficiency by fine-tuning the diffusion coefficient and concentration gradient of redox substances within the ionic gel as shown in Fig. 9f. EtV ^2+^ serves a dual role as an electrochromic material and a redox-active substance within supercapacitors, endowing the device the ability of real-time energy-storing status in color intensity. Crucially, the performance of the supercapacitor was significantly enhanced, achieving a top-level energy density (~ 43.0 mF cm^−2^), by enhancing the diffusion flux of the GPE through controlled manipulation of both the diffusion coefficients and concentration gradients of the redox species. Electrochromic supercapacitors with additional self-healing properties are attractive for flexible wearable devices. Guo et al. prepared electrochromic supercapacitors based on self-healing hydrogel electrolytes, which can be used normally after bending, stretching or even cutting [173]. Due to the abundant hydrogen bonding in PVA, GPEs have good stretchability and can be self-healing within 4 h at room temperature. The supercapacitor device has a face capacitance of up to 61 mF cm^−2^ and excellent self-healing properties, laying the groundwork for the development of future wearable devices.

**Fig. 10:**
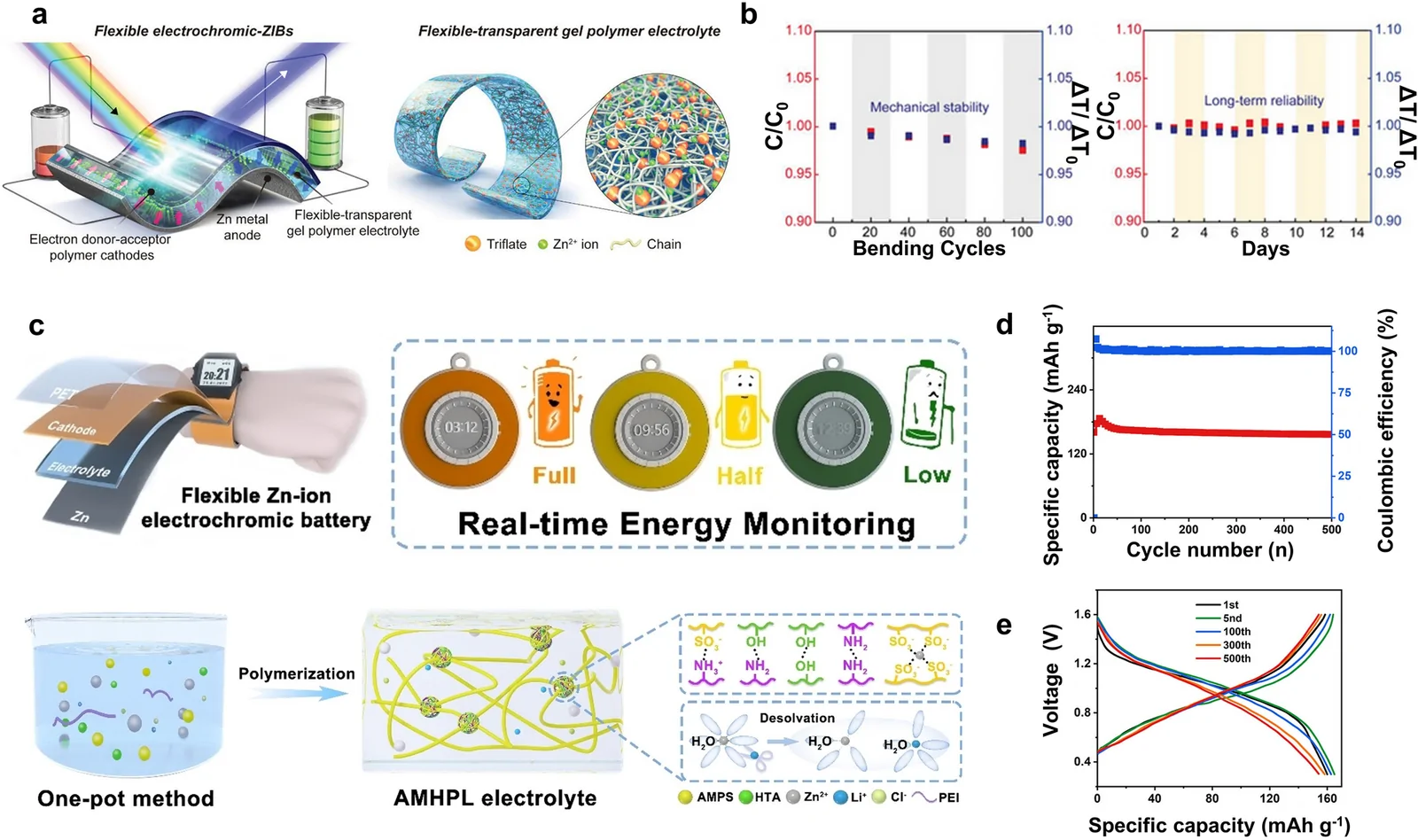
Application of GPEs in electrochromic batteries. **a** Schematic representation of flexible electrochromic-ZIBs and PMMA-based GPEs. **b** Bending stability and long-term stability of flexible electrochromic ZIBs. Reproduced with permission [174]. Copyright 2023, Wiley–VCH Verlag.**c** Structure and principle of flexible electrochromic ZIBs and GPEs. **d** and **e** Cycle stability of electrochromic ZIBs. Reproduced with permission [175]. Copyright 2024, Wiley–VCH Verlag

Compared to supercapacitors, batteries are extensively utilized due to their higher energy density [163]. GPEs are extensively applied in electrochromic batteries as well. As illustrated in Fig. 10a, Il-Doo Kim et al. fabricated an electrochromic zinc-ion battery (ZIBs), which encompasses an electron donor–acceptor polymer cathode/electrochromic layer, Zn–metal anode and PMMA GPEs [174]. The application of PMMA-based GPEs facilitated the absorption of aprotic electrolytes, prevented leakage or evaporation of the electrolyte, and concurrently provided excellent mechanical support for the fabrication of free-standing batteries. Owing to the remarkable mechanical strength and stability of the PMMA GPEs, the electrochemical and electrochromic properties of the electrochromic zinc–ion battery exhibited negligible deterioration after multiple bending cycles and a 14-day durability test (Fig. 10b), demonstrating the mechanical stability and long-term durability of the device. Most current electrochromic energy storage devices can merely achieve a single color intensity alteration, and it is arduous to indicate the energy storage level through multiple continuously variable colors. As presented in Fig. 10c, Yan et al. have designed a flexible zinc–ion electrochromic cell with reversible multicolor conversion (from orange to brown to green) [175]. The zinc–ion cell was assembly with multicolor electrochromic sodium vanadate nanorods cathode, flexible GPE and Zn anode. The anchored − SO_3_^−^ and − NH_3_^+^ groups in GPE as ion redistributor contributes to the formation of ionic transportation channels for enhancing Zn^2+^ transportation kinetics and promoting the oriented Zn^2+^ deposition on Zn (002), thus inhibiting the generation of Zn dendrite. Figure 10d, e shows that the electrochromic zinc–ion battery exhibits high capacity, long cycle life and reversible multi-color transition (orange-brown-green) for real-time monitoring of energy storage, which provides significant advantages in the zinc–ion battery system.

At present, most of the electrochromic batteries are committed to modulation of visible light, while the electrochromic batteries with good modulation performance for infrared wavelengths are still relatively few. Zhi et al. prepared reflective electrochromic batteries with infrared tunability using hydrogel electrolytes possessing proton and iron ion conductivity and cross-linked polyaniline as core materials [176]. Co-conduction of protons and complexed iron ions in the hydrogel electrolytes provides access to fast reaction kinetics and enhanced electrochemical performance for the electrochromic batteries. The electrochromic cell has stable complex Fe(TOF) and H cost storage properties, delivering excellent long cycle life (107 mAh g^−1^ after 39,000 cycles at 25 A g^−1^) and high rate performance (120 mAh g^−1^ at 25 A g^−1^). In addition, the reflective electrochromic cell has excellent infrared modulation performance, which has the potential to be used in spacecraft thermal control or camouflage applications.

### Application of GPEs in Electrochromic Displays

Displays are particularly relevant to human life as the interface for smart electronics, conveying visual information and interaction [177]. The working principle of electrochromic displays is based on the electrochemically driven redox process of electrochromic materials, thereby generating variations in optical absorption rate, transmittance or reflectance for presenting display content and information [5]. Compared to other display technologies, electrochromic displays have obvious advantages such as low power consumption, easy viewing, high contrast and easy flexibility [178, 179]. With the rapid development in the field of optoelectronics, electrochromic displays are gaining more and more attention and show attractive application potential in emerging wearable and portable electronics, e-paper, billboard and other new generation displays [5, 180]. GPEs with high ionic conductivity and mechanical flexibility are expected to enable faster switching speeds for electrochromic displays and broaden their application in wearable facilities.

**Fig. 11:**
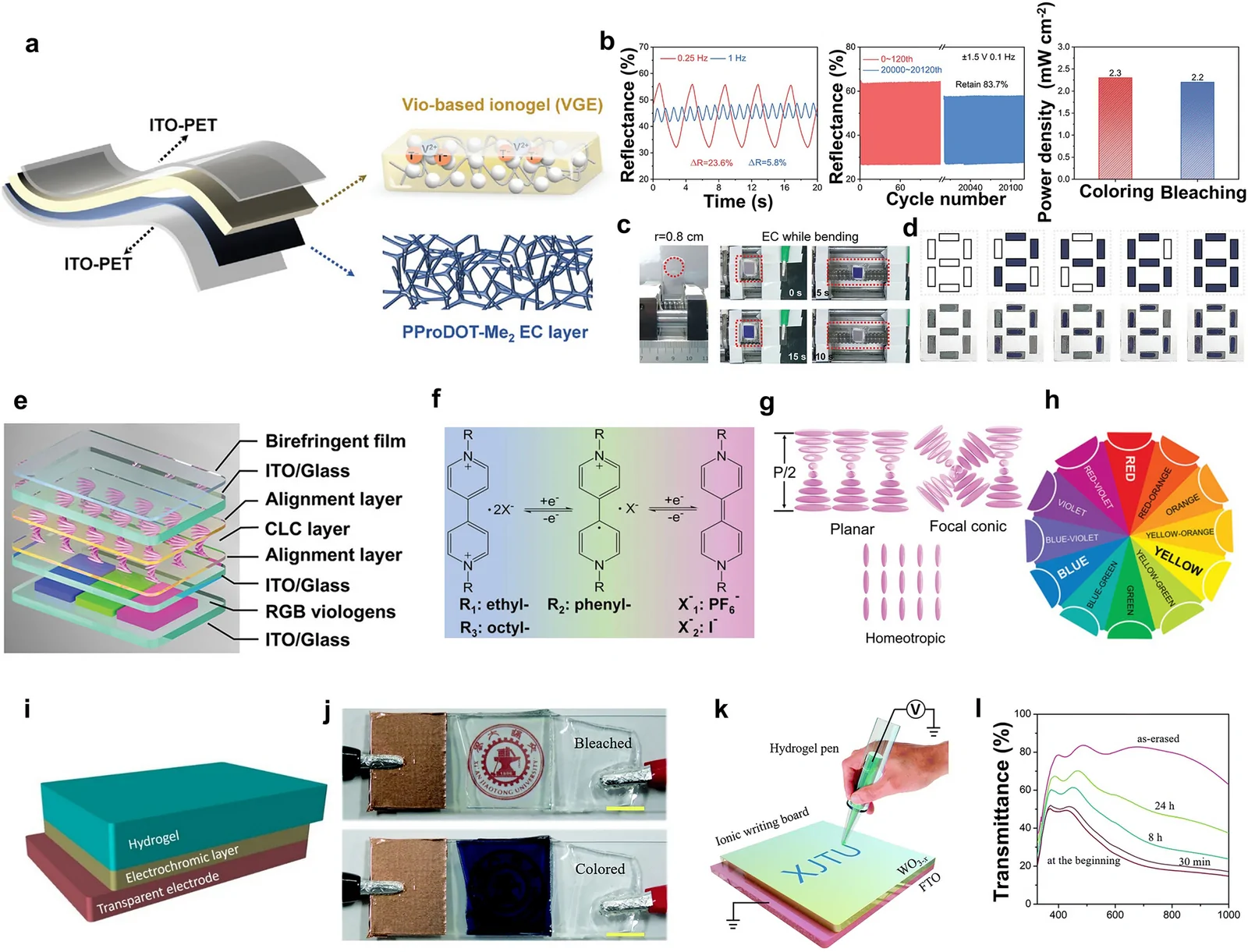
Application of GPEs in electrochromic displays. **a** Structure of flexible electrochromic displays. **b** Switching speed, cycling stability and power density of the flexible electrochromic displays. **c** Bending stability of the flexible electrochromic displays. **d** Schematic diagrams and digital photographs of the electrochromic display (showing numbers: 0, 2, 4, and 6). Reproduced with permission [181]. Copyright 2023, Wiley–VCH Verlag. **e** Structure of the multi-color electrochromic display. **f** Structure and electrochromic effect of different substituted viologens. **g** Schematic diagram of three types of rod-shaped liquid crystal structures. **h** Multiple color display modes achieved by combined liquid crystals with viologens-based GPEs. Reproduced with permission [97]. Copyright 2023, Wiley–VCH Verlag. **i** Structure of the GPE-based electrochromic handwriting displays. **j** Digital photograph of the electrochromic effect of the devices. **k** Schematic diagram illustrating the handwritten coloring principle and self-erased performance of the devices. **l** Self-bleaching performance of the devices. Reproduced with permission [182]. Copyright 2018, Royal Society of Chemistry

The electrochromic e-paper technology, adopting the reflective display principle similar to that of traditional books, offers significant advantages in terms of enhanced reading comfort and reduced energy consumption. As shown in Fig. 11a, Wang et al. prepared a white divalent viologen cation-based ionogel and assembled the flexible electrochromic e-paper with poly (3,4-(2,2-dimethylpropylenedioxy)thiophene) (PProDOT-Me_2_) [181]. Divalent viologen cation in the ionogel electrolyte provided more reaction charges and as an electrochromic cation can also color on the back of the electrochromic e-paper. Figure 11b shows that the device exhibited a rapid switching speed (coloring/bleaching time is 2.9/3.3 s), robust cycling stability (maintain 83.7% after 20,000 cycles) and low operating energy consumption (coloring/bleaching energy consumption is 2.3/2.2 mW cm^−2^). In addition, the device can operate stably at a curvature radius of 0.8 cm, which possesses great application potential in the field of flexible e-paper as shown in Fig. 11c. In addition, the device can also be assembled into an 8-bit electrochromic display and numbers from 0 to 9 were displayed through individual control of each component (Fig. 11d). Electrochromic displays with multi-color adjustability are more appealing than those that can only display a single color. By compounding two viologens, monoheptyl viologen (MHV^+^) and diheptyl viologen (DHV^2+^), into an ion-GPE, Hong Chul Moon's team prepared an all-in-one integrated electrochromic display with multicolor tunability [31]. The resulting electrochromic displays showed diverse colors when the applied voltage was judiciously adjusted: slightly yellowish (bleached state), blue (colored state I at − 0.8 V) and maroon (colored state II at − 1.3 V). In order to achieve more colors in electrochromic displays, liquid crystals are incorporated into ECDs. As shown in Fig. 11e, Liu et al. proposed an electrochromic display with enlarged color palette and multi-mode color modulation by integrating liquid crystals with the viologens ionogel electrolyte-based ECD [97]. Three substituents of dioctyl-, diphenyl-, and diethyl- were integrated with dipyridyl to generate red, green, blue colors, respectively (Fig. 11f). Three alignments of planar, focal conic and homeotropic states of rod-shaped liquid crystal molecules as shown in Fig. 11g can be reversibly converted to each other under an applied voltage, thus realizing reversible switching between transparent and hazy states in electrochromic displays. By adjusting the hue, saturation and brightness of the three primary colors, a variety of colors of this colorized electrochromic display were produced (Fig. 11h), which had great potential for application.

The display content of traditional electrochromic displays is predetermined by the patterning of the electrode, electrochromic material or electrolyte, signifying that the display information cannot be updated once the device is fabricated. As shown in Fig. 11i, j, Wang et al. prepared a multifunctional polyacrylamide hydrogel containing lithium chloride and utilized the hydrogel as an electrolyte layer for electrochromic displays, an exemplary storage layer and a transparent electrode [182]. The hydrogel can be injected into the WO_3-x_ thin film by handwriting (Fig. 11k), resulting in a localized electrochromic structure. The device showed a slow self-bleaching effect, enabling the written pattern to exist for several hours without energy consumption as shown in Fig. 11l. The device can realize local erasing and repeated writing of patterns and has great application prospects in the field of electrochromic display.

GPEs with stretchability and self-healing properties contribute to the durability of electrochromic displays. Matsuhiko Nishizawa et al. realized stretchable electrochromic displays by preparing electrochromic materials and hydrogel electrolytes with stretchability [183]. The display does not require an additional conductive substrate and has good electrochromic properties even under stretching, thus allowing it to be used as a display for wearable devices. Xu et al. prepared customizable patterned electrochromic displays based on freeze-resistant self-healing organic hydrogels [184]. The hydrogel electrolyte is cross-linked by dynamic covalent Schiff base bonds, resulting in excellent self-healing and injectable properties. Thanks to the high reversibility of dynamic covalent bonding, the hydrogel electrolyte possesses excellent self-repairing properties and can completely heal the severed gel within 240 s. Self-healing GPEs greatly improve the durability of electrochromic displays and therefore have potential for practical applications.

### Application of GPEs in Electrochromic Wearable Devices

Wearable ECDs exhibit significant potential for next-generation electronics, including electronic skins, adaptive camouflage, flexible displays and human–machine interfaces [1, 53, 185–187]. To fully exploit the performance of wearable electronic devices, electrochromic devices should be flexible and capable of presenting good possible electrochromic performance under various deformations (including stretching, compression, bending, etc.) during daily activities [188, 189]. Conventional liquid electrolytes tend to be unevenly distributed within the ECD under external deformation stress and can potentially lead to leakage, which can seriously compromise the reliability and safety of wearable ECDs [166]. GPEs, which combines high ionic conductivity, good mechanical strength and flexibility, can maintain stability under deformation of ECDs, making them an ideal electrolyte material for wearable ECDs. Additionally, the components of ECDs are tightly connected together during deformation due to the intrinsic adhesion and elasticity of GPEs, which avoids delamination failure of the device.

**Fig. 12:**
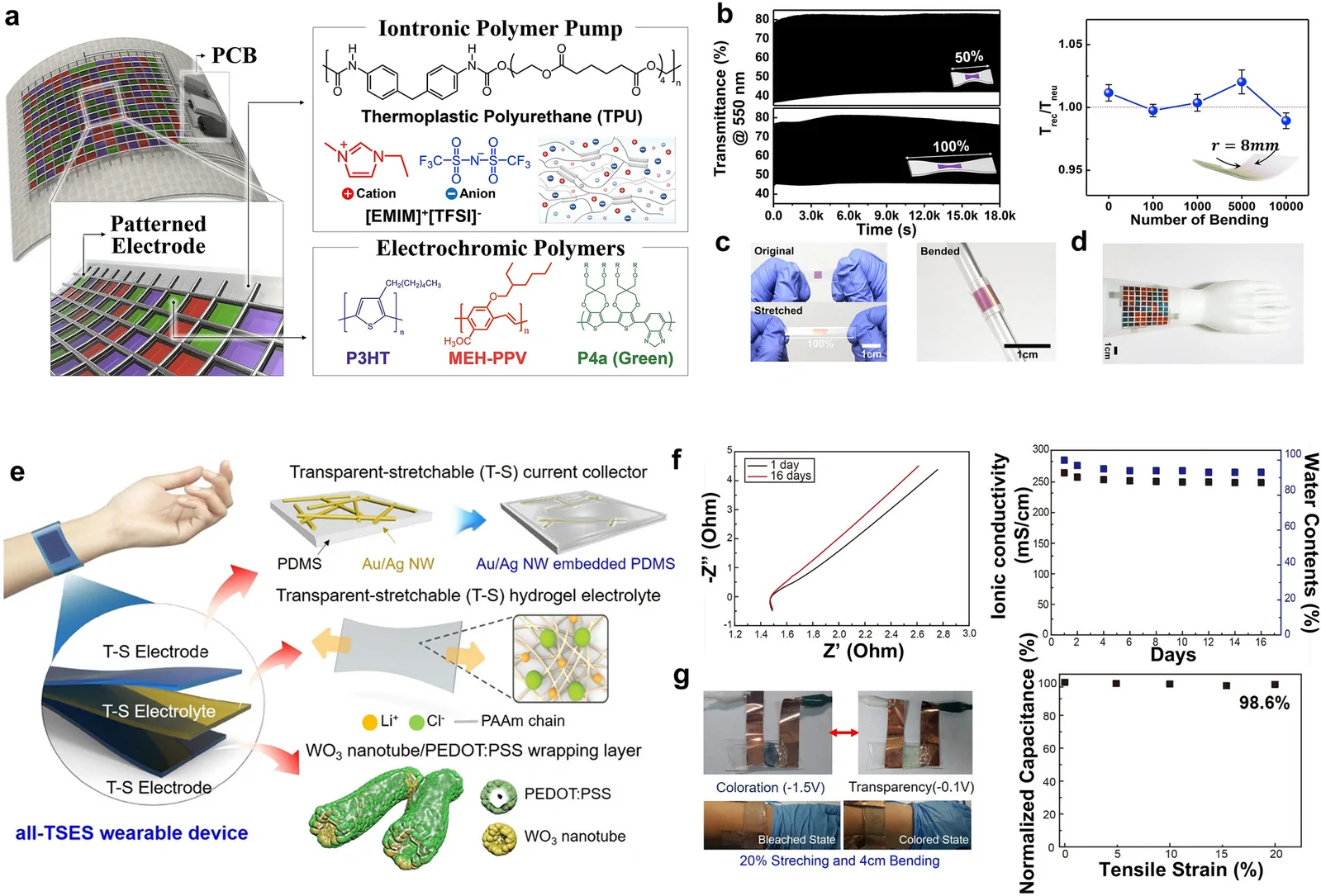
Applications of GPEs in wearable electrochromic skin. **a** Structure of the wearable, low-power, dynamic multicolor electrochromic skins. **b, c** Electrochromic properties of the electrochromic skin under tension and bending. **d** Image of the integrated electrochromic skin. Reproduced with permission [192]. Copyright 2020, Elsevier.** e** Structure of wearable electrochromic supercapacitors. **f** Long-term stability of GPEs. **g** Performance of wearable electrochromic supercapacitors in the stretched state. Reproduced with permission [166]. Copyright 2019, American Chemical Society

Electrochromic skin is a pivotal application of wearable ECDs, playing crucial roles in domains such as active camouflage, smart displays and thermal regulation [185, 190, 191]. Do Hwan Kim et al. developed a wearable, low-power, dynamic multicolor electrochromic skin based on the deformable transparent ionogel electrolyte and three different electrochromic polymers as depicted in Fig. 12a [192]. GPEs with optimal polymer-IL ratios were fabricated by balancing optical transparency, mechanical strength and ionic conductivity. As presented in Fig. 12b, c, owing to the ideal performance of the GPEs, the device remains stable over long periods under both stretching and bending, demonstrating that the device has potential for wearable applications. Additionally, the device possesses the advantage of being integrable over a large area as well as low power consumption, opening up new avenues for potential applications in human adaptive camouflage and multicolor wearable displays (Fig. 12d). For wearable electrochromic skins, aging or long-term bending can cause unavoidable damage to the devices. Self-healing ECDs prevent such damage to a large extent, thereby increasing the durability of the devices. Jia et al. proposed a self-healing electrochromic film and GPE-based electrochromic skin. The researchers first prepared a copolymer film with biomimetic properties that incorporates both electrochromic triphenylamine and self-healing Diels–Alder groups [193]. Combined with GPEs with excellent ionic conductivity and mechanical properties, the ECDs exhibited excellent electrochromic properties while demonstrating excellent self-healing efficiency (90%).

Conventional electrochromic devices are difficult to disengage from external power sources, which is inconvenient for wearable applications of the devices. The ECDs, which can also be combined with energy storage devices, are anticipated to be self-sufficient without relying on external power sources, making them highly desirable for wearable devices. As shown in Fig. 12e, Il-Doo Kim's team prepared a wearable electrochromic supercapacitor by developing and integrating the transparent stretchable GPE and collector [166]. In addition to excellent stretchability, the hydrogel electrolyte had excellent water retention capability (Fig. 12f), which perfectly solved the problem of electrolyte evaporation in the device and leakage during mechanical deformation. The device still exhibited good electrochromic effects under stretching and bending and had a capacitance retention of 98.6% at 20% total strain as shown in Fig. 12g, indicating that it is well suited for wearable devices. To further improve the adaptability of wearable ECDs to diverse shapes, Seok Woo Lee's team developed an ultrathin electrochromic energy storage device for skin-interfaced wearable electronics via a simple and scalable transfer printing approach [194]. The free-standing submicron thick cathode film of the device was initially fabricated by coating on a glass plate and subsequently easily detached from the glass plate by a capillary-assisted layering process and transferred onto a hydrogel electrolyte grafted onto a silanized zinc anode. Owing to its high flexibility and mechanical stability, the device can be customized into arbitrary shapes and exhibits excellent conformability on irregular surfaces, which provides an avenue for the development of multifunctional electronic skins and next-generation wearable electronics.

**Fig. 13:**
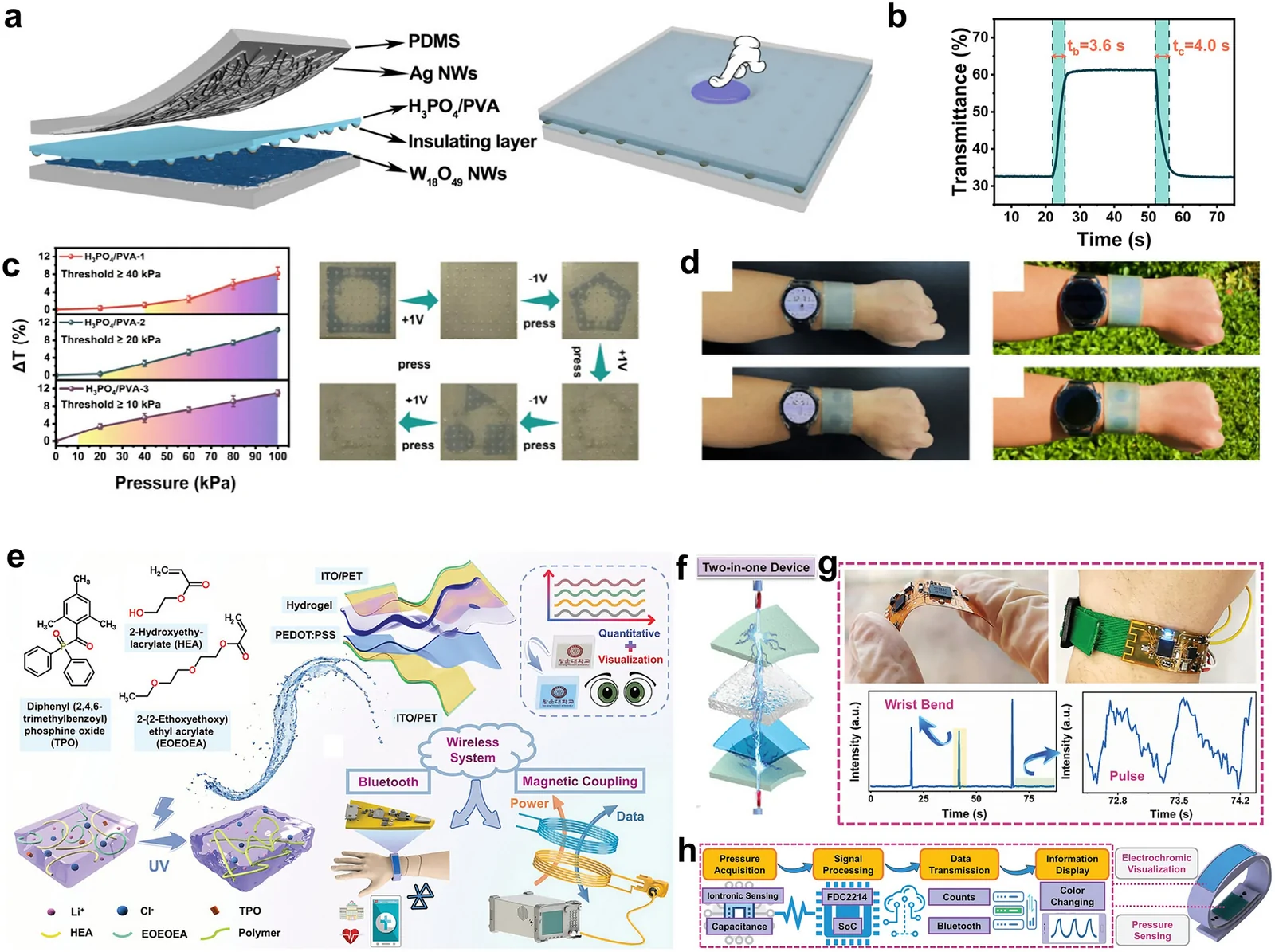
Applications of GPEs in wearable electrochromic sensor. **a** Schematic diagram of electrochromic pressure sensor structure. **b** Electrochromic performance and response time of the device. **c** Visualize sensing performance of the device. d Comparison of display performance between the device and traditional light-emitting devices under strong light. Reproduced with permission [203]. Copyright 2025, American Chemical Society. **e** Structure, principle and working mode of the integrated wearable electrochromic sensor. **f** Schematic diagram of the structure of a two-in-one electrochromic sensor. **g** Images of the fabricated flexible system worn on the wrist, and its continuous monitoring data.** h** Schematic diagram of the workflow of the electrochromic sensors. Reproduced with permission [204]. Copyright 2023, Wiley–VCH Verlag

In recent years, there has been an increasing interest in physiological monitoring through devices interfacing with the human skin or organs, as evidenced by the escalating number of research and development activities in this area [195–198]. The establishment of wearable sensor systems aimed at detecting and quantifying physical and chemical signals from the human body offers promising opportunities for disease diagnosis, treatment and health management [197, 199, 200]. Additionally, wearable ECDs are frequently integrated into sensors to deliver visual sensing outputs [201, 202]. Nevertheless, current wearable sensor technologies typically rely on external devices for power and data visualization, which restricts their practicality and ease of utilization. In order to overcome the dependence of wearable electrochromic sensors on external devices, Joseph Wang et al. reported on a wearable integrated skin sensing platform with an integrated electrochromic display for real-time visualization of analytical data and high-performance stretchable battery power [202]. The skin patch consists of a stretchable enzyme and potential electrochemical sensor for sensing various metabolites and electrolytes in sweat, a stretchable battery, a low-power electrochromic display and an integrated controller chip. GPEs with stretchable properties were used in ECD and battery of the device to prevent electrolyte leakage during stretching and bending. Furthermore, the electrical connections between the system components are printed using a stretchable silver ink layer treated with chlorine/lactic acid, thus demonstrating a stable electrical connection under repeated stretching. This fully autonomous, multifunctional, self-sustainable wearable sweat sensing platform has exceptional applications in personal health management, medical monitoring and professional sports. Wearable electrochromic sensors also have a wide range of applications in mechanosensing. As shown in Fig. 13a, Yu et al. prepared an electrochromic pressure sensing device with in situ visualization of pressure information [203]. The ECDs have excellent optical modulation and fast response time, ensuring timely visual sensing (Fig. 13b). Further, the device deepens in color as the pressure increases and the process is reversible, thus demonstrating the viability as an in situ visual pressure sensing (Fig. 13c). As can be seen from Fig. 13d, the pressure sensor based on electrochromic technology still has a good visual effect under strong light, which has a significant advantage over light-emitting devices. The integrated devices developed in the aforementioned study are interconnected and packaged alongside devices with diverse functionalities, leading to challenges related to complex device structures and intricate preparation processes. In a separate study, Jong-Chul Lee's team utilized a photocurable ionic hydrogel to develop a two-in-one wearable device that integrates ionic electronic pressure sensing and electrochromic display (Fig. 13e), effectively streamlining the complexity of the device and manufacturing process [204]. As shown in Fig. 13f, compared with the typical stack of multiple independent devices, the proposed two-in-one device reduced the manufacturing complexity by sharing the functional layer between the electrochromic device and the ionic-electronic pressure sensor. In addition, this strategy combines Bluetooth technology and magnetic coupling effects to achieve multiple methods of pressure sensing information transmission as presented in Fig. 13g, h, offering significant potential for flexible electronic products and multifunctional sensing platforms.

In this section, we focus on the practical applications of these devices and summarize the practical applications of GPEs in electrochromic smart windows, electrochromic energy storage apparatuses, electrochromic displays and wearable electrochromic devices. Electrochromic devices based on GPEs have exhibited exceptional performance across different application scenarios, which fully attests to the prominent advantages of GPEs as the electrolyte layer for electrochromic devices. Endowed with remarkable ion-transport properties, mechanical stability and versatile designs, GPEs demonstrate substantial potential in the realm of practical applications of electrochromic devices, thereby establishing themselves as the ideal electrolyte material for such devices.

## Conclusions and Perspectives

### Conclusion

In this review, we provide a comprehensive overview rom three aspects: the basics of GPEs for ECDs, functionalized design and finally to practical applications. Drawing on recent research findings regarding GPEs for ECDs, this review firstly presents a comprehensive overview of their physical and electrochemical properties, classification, gelation mechanisms and preparation methods. Subsequently, it places a strong emphasis on the development of functionalized GPEs designed to meet the ever-evolving requirements of ECDs, including intrinsically electrochromic, temperature-responsive, photo-responsive, self-healing and stretchable GPEs. Furthermore, this review meticulously details the design strategies of ECDs based on GPEs and their applications across a diverse range of scenarios. The overarching objective is to elucidate how the rational design of GPEs can drive the research and development of high-performance ECDs that are adaptable to a wide array of practical applications.

### Challenges and Solutions

**Fig. 14:**
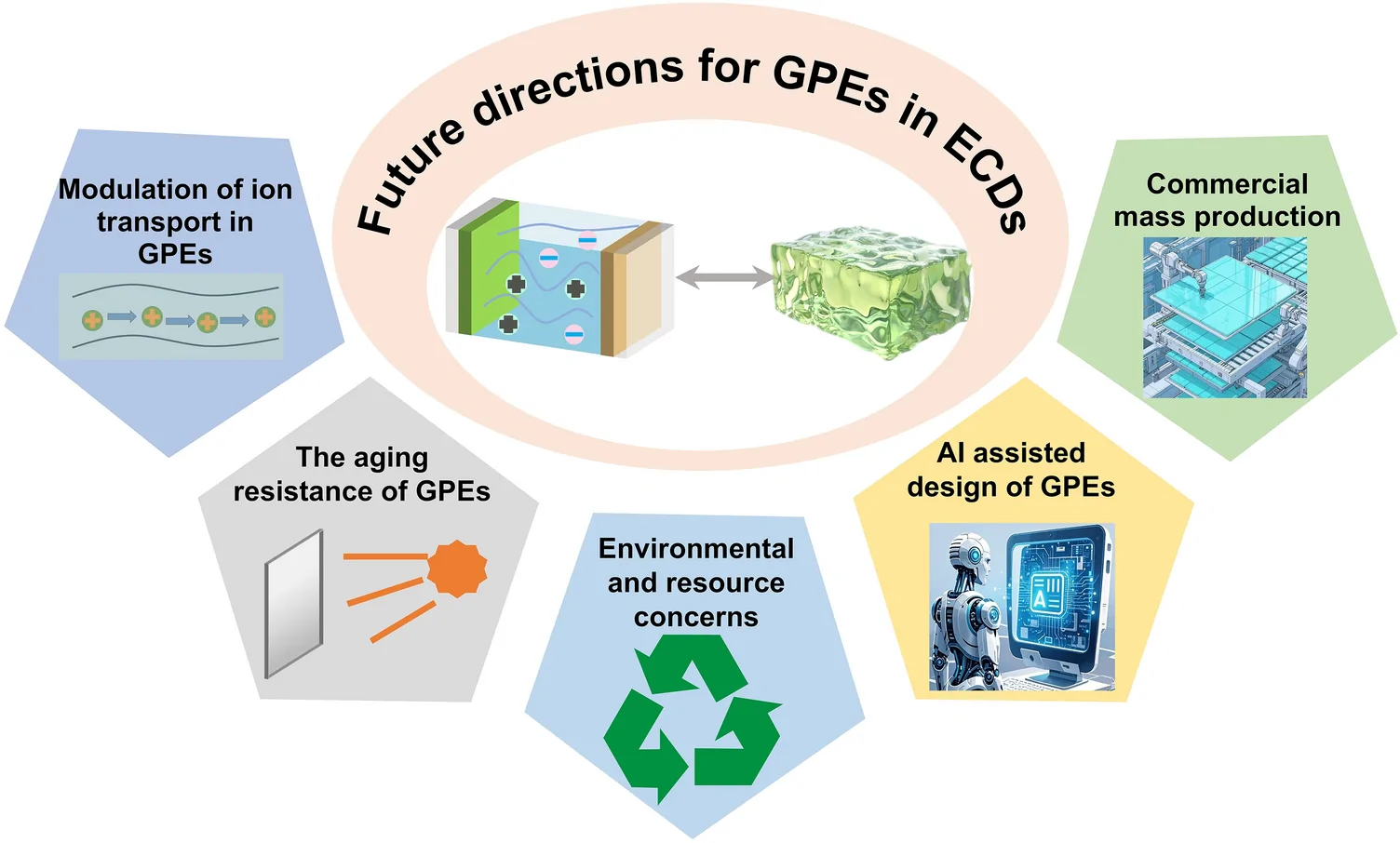
Prospective development orientation of GPEs for ECDs

Although significant progress has been achieved in the development of GPEs for ECDs, several challenges remain to be addressed. Furthermore, for the successful commercialization of GPE-based ECDs, it is imperative to direct greater attention toward scalable manufacturing processes and the selection of materials that offer high cost-effectiveness. As depicted in Fig. 14, this review highlights several challenges that remain underexplored, which may offer valuable direction for future research and the practical implementation of GPEs for ECDs.

*Modulation of ion transport in GPEs:* Ion transport is the most fundamental function of GPEs as electrolytes. However, current GPEs for ECDs still lack effective modulation strategies for ion transport. The metal cations of GPEs are in complex solvated structures with their anions or plasticizers and polymers. The more polar solvents are good for facilitating the dissociation of the salts, but have a strong binding effect on the metal cations making them difficult to desolventize. Therefore, the solvated structures in GPEs need to be modulated to achieve both efficient dissociation of metal salts as well as desolvation of metal cations for efficient ion transport. For electrochromic devices, a high lithium ion transference number approaching 1 is urgently required. However, due to the stronger interaction of the polymer chains and plasticizers on the metal cations, the migration of the metal cations is significantly weaker than that of the anions, thus adversely affecting the performance of the ECDs. Based on this, there is a need to consider introducing strategies to limit anion migration in GPEs. The design of the polymer chains to improve the interaction with the anions or directly anchoring the anions to the polymer chains through covalent bonding will effectively increase the mobility number of the metal cations in the GPEs.

*The aging resistance of GPEs:* ECDs are predominantly utilized outdoors, and GPEs are unavoidably exposed to sunlight for prolonged durations. This demands that GPEs possess excellent aging resistance to avert issues such as yellowing and substantial performance attenuation resulting from polymer chain rupture. Therefore, for the preparation of GPEs for ECDs, polymers with poor UV aging resistance need to be avoided, and the addition of components like UV absorbers needs to be considered. Additionally, GPEs circumvent the safety hazards associated with electrolyte leakage; however, if the encapsulation is cracked, external air and water can likewise exert adverse influences on GPEs. Some ILs are inherently hydrophobic and oxygen-phobic, which can effectively improve the stability of GPEs. In addition, because of the high ionic conductivity of the ILs itself, the use of metal salts can sometimes be avoided, further improving the stability of GPEs. At present, although research related to GPEs for ECDs mainly centers on the cycling stability (lifespan) of ECDs, these tests are carried out under laboratory conditions, disregarding the influence of the external environment on the devices. Consequently, the design of GPEs with aging resistance and stability and the assessment of related outdoor performance are of vital importance for the practical application of ECDs.

*Environmental and resource concerns:* The processing cost of ECDs and the post-scrapping treatment represent significant issues in their practical application. In the actual application of ECDs, the damage to the devices is typically caused by the limited lifespan of the electrochromic materials rather than problems with GPEs. At this juncture, GPEs can be fully recovered from the scrapped electrochromic devices and reemployed in new electrochromic devices. Even if the performance of GPEs has severely deteriorated or failed, the effective components therein can still be extracted and reused. The adoption of a recycling and reuse method will conspicuously reduce the production cost of ECDs and concurrently lower environmental pollution. Hence, how to achieve the recycling and utilization of GPEs is also a direction that demands key attention in the future.

*Artificial intelligence (AI) assisted design of GPEs:* The development of GPEs is a tedious process involving extensive material design, screening and performance testing. So, the development of GPEs for ECDs takes a lot of time and cost. AI is capable of rapidly screening out electrolyte systems with potential performance advantages by analyzing large amounts of experimental data and theoretical calculations. It can further predict the influence of diverse molecular structures on the performance of electrolytes based on the known electrolyte performance data, thereby guiding the design of molecular structures for the main materials of GPEs with superior performance. By means of the prediction and simulation of artificial intelligence, researchers can expeditiously assess the feasibility of different design schemes, reduce the number of experiments and economize time and costs. Consequently, the integration of artificial intelligence with the design of GPEs constitutes one of the future development trends.

*Commercial-oriented large-area and large-scale preparation industry:* Currently, the preparation process of GPEs utilized in ECDs is relatively mature, however, numerous preparation methods remain at the laboratory stage. To fulfill commercialization requirements, attaining a preparation process capable of rapid, large-scale and batch production is of paramount importance. For ECDs that are temporarily difficult to large area, consider integrating multiple small devices together. Simultaneously, attention should also be directed toward the homogeneity and pass rate of large-scale production of GPEs to prevent local defects that might lead to the failure of ECDs.

In conclusion, GPEs as the core materials of ECDs exhibit extensive functionality and adaptability. Their development not only propels the progress of electrochromic technology but also pioneers abundant possibilities for innovation in the domain of smart materials. Looking into the future, as materials science and preparation techniques continue to evolve, the application of GPEs in electrochromic devices is anticipated to make more breakthroughs and undergo broader expansions.
